# Shoot differentiation from protocorm callus cultures of *Vanilla planifolia *(Orchidaceae): proteomic and metabolic responses at early stage

**DOI:** 10.1186/1471-2229-10-82

**Published:** 2010-05-05

**Authors:** Tony L Palama, Patrice Menard, Isabelle Fock, Young H Choi, Emmanuel Bourdon, Joyce Govinden-Soulange, Muriel Bahut, Bertrand Payet, Robert Verpoorte, Hippolyte Kodja

**Affiliations:** 1 UMR "Peuplement végétaux et Bioagresseurs en Milieu Tropical" Faculté des Sciences et Technologies, Université de La Réunion, 15 Avenue René Cassin, Saint-Denis, La Réunion, France; 2 Division of Pharmacognosy, Section Metabolomics, Institute Biology, Leiden University, Leiden, The Netherlands; 3 Laboratoire de Biochimie et Génétique Moléculaire, Faculté des Sciences et Technologies, Université de La Réunion, 15 Avenue René Cassin, Saint-Denis, La Réunion; 4 Faculty of Agriculture, University of Mauritius, Réduit, Mauritius; 5 Plate Forme de Biotechnologies moléculaires, Faculté des Sciences, Université d'Angers, 22, rue Roger Amsler, 49100 Angers, France; 6 Laboratoire de Chimie des Substances Naturelles et des Sciences des Aliments, Faculté des Sciences et Technologies, Université de La Réunion, 15 Avenue René Cassin, Saint-Denis, La Réunion, France

## Abstract

**Background:**

*Vanilla planifolia *is an important Orchid commercially cultivated for the production of natural vanilla flavour. Vanilla plants are conventionally propagated by stem cuttings and thus causing injury to the mother plants. Regeneration and *in vitro *mass multiplication are proposed as an alternative to minimize damage to mother plants. Because mass production of *V. planifolia *through indirect shoot differentiation from callus culture is rare and may be a successful use of in *vitro *techniques for producing somaclonal variants, we have established a novel protocol for the regeneration of vanilla plants and investigated the initial biochemical and molecular mechanisms that trigger shoot organogenesis from embryogenic/organogenic callus.

**Results:**

For embryogenic callus induction, seeds obtained from 7-month-old green pods of *V. planifolia *were inoculated on MS basal medium (BM) containing TDZ (0.5 mg l^-1^). Germination of unorganized mass callus such as protocorm -like structure (PLS) arising from each seed has been observed. The primary embryogenic calli have been formed after transferring on BM containing IAA (0.5 mg l^-1^) and TDZ (0.5 mg l^-1^). These calli were maintained by subculturing on BM containing IAA (0.5 mg l^-1^) and TDZ (0.3 mg l^-1^) during 6 months and formed embryogenic/organogenic calli. Histological analysis showed that shoot organogenesis was induced between 15 and 20 days after embryogenic/organogenic calli were transferred onto MS basal medium with NAA (0.5 mg l^-1^). By associating proteomics and metabolomics analyses, the biochemical and molecular markers responsible for shoot induction have been studied in 15-day-old calli at the stage where no differentiating part was visible on organogenic calli. Two-dimensional electrophoresis followed by matrix-assisted laser desorption ionization time-of-flight-tandem mass spectrometry (MALDI-TOF-TOF-MS) analysis revealed that 15 protein spots are significantly expressed (*P *< 0.05) at earlier stages of shoot differentiation. The majority of these proteins are involved in amino acid-protein metabolism and photosynthetic activity. In accordance with proteomic analysis, metabolic profiling using 1D and 2D NMR techniques showed the importance of numerous compounds related with sugar mobilization and nitrogen metabolism. NMR analysis techniques also allowed the identification of some secondary metabolites such as phenolic compounds whose accumulation was enhanced during shoot differentiation.

**Conclusion:**

The subculture of embryogenic/organogenic calli onto shoot differentiation medium triggers the stimulation of cell metabolism principally at three levels namely (i) initiation of photosynthesis, glycolysis and phenolic compounds synthesis; (ii) amino acid - protein synthesis, and protein stabilization; (iii) sugar degradation. These biochemical mechanisms associated with the initiation of shoot formation during protocorm - like body (PLB) organogenesis could be coordinated by the removal of TDZ in callus maintenance medium. These results might contribute to elucidate the complex mechanism that leads to vanilla callus differentiation and subsequent shoot formation into PLB organogenesis. Moreover, our results highlight an early intermediate metabolic event in vanillin biosynthetic pathway with respect to secondary metabolism. Indeed, for the first time in vanilla tissue culture, phenolic compounds such as glucoside A and glucoside B were identified. The degradation of these compounds in specialized tissue (i.e. young green beans) probably contributes to the biosynthesis of glucovanillin, the parent compound of vanillin.

## Background

The genus *Vanilla *belongs to the family Orchidaceae, which consists of more than 110 described species [1]. *Vanilla planifolia *Andrews, *V. tahitensis *and *V. pompona *are the commercially important species cultivated for the production of natural vanilla flavour [2]. *Vanilla planifolia *is preferred to the two other species for its flavour [3,4].

The seeds of vanilla do not usually germinate and hence the plants are propagated by vegetative means through stem cuttings resulting in a slow rate of multiplication. Moreover, this method is not economical since the collection of stem cuttings leads to the interruption of growth of the mother plant, resulting in a yield reduction which can be minimized by *in vitro *regeneration and mass multiplication. Hence, the *in vitro *multiplication of *V. planifolia *using shoot tip and axillary buds has been frequently reported [5,6]. Thus cultivated plants are genotypically identical but all could be sensitive to the aggressiveness of virulent soil pathogens [7]. Somaclonal variation can be induced if regeneration includes callus culture [8]. Unfortunately plantlet regeneration from callus of *V. planifolia *has not been reported so far [9]. Mass production of *V. planifolia *through indirect shoot organogenesis is rare and there are numerous biological unknowns about the induction of this morphogenesis.

Organogenesis and somatic embryogenesis are both morphogenetic processes leading to plantlet regeneration. It is postulated that control over embryogenic development occurs at the protein and metabolite levels [10]. Proteomic approaches have been applied to investigate the protein profiles and their changes during callus differentiation [11,12] and induction of somatic embryogenesis [13-16]. All these studies have applied the two-dimensional electrophoresis (2-DE) technique which is often employed for the analysis of differential protein expression across biological samples [17]. Moreover, proteomic approaches were achieved using both 2-DE technique for protein separation and MALDI-TOF-TOF-MS for protein identification [11,12,15,18].

Metabolomics approaches have also been applied to study white spruce somatic embryogenesis [10]. The ultimate goal of metabolomics is both qualitative and quantitative analysis of all metabolites in an organism or tissue [19]. It can provide a broad view of the biochemical status of an organism in interaction with its environment. Chemical analysis techniques applied to metabolomic profiling involves Nuclear Magnetic Resonance spectroscopy (NMR) since NMR can provide direct molecular structure information in the analysis of complex biological mixtures such as cell growth media [10] or plant extracts [20,21].

Proteomic and metabolomic analysis using NMR techniques can be considered as two complementary techniques. As such, proteomic analysis highlights changes in the more abundant proteins, including many enzymes whereas NMR analysis reveals differences in the more abundant metabolites. Therefore, metabolomics will provide crucial information for a functional interpretation of proteomic data, while proteomic analysis contributes to a better understanding of metabolomic data by highlighting the enzymes or enzymatic pathways involved [22]. Consequently, a combination of these methods represents great potential for exploring the biological processes involved in response to environmental changes such as the effect of plant hormones in culture medium. In this study, by associating proteomics and metabolomics, we have used organogenic calli from *V. planifolia *protocorm to investigate their metabolic and protein profiles and their changes during earlier stages of shoot differentiation. We have identified several proteins whose expression differed during early events of shoot organogenesis. In addition, some metabolites were also characterized regarding this morphogenesis and subsequent *Vanilla *plant regeneration.

## Results

### Adventitious shoots and in vitro plant formation

**Figure 1:**
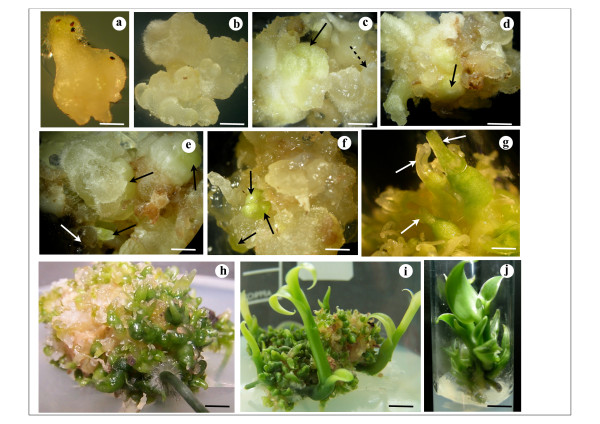
**Plant regeneration from protocorm callus of *V. planifolia***. **a**. Protocorm-like structure (PLS) from seed on germination medium; bar = 1.5 mm. **b**. Embryogenic callus from PLS after transfer to callus induction medium; bar = 1 mm. **c**. Embryogenic/organogenic callus mass showing nodular compact structure (dashed arrow) and pale green zone (arrow) after 30 days of culture on A4 medium (i.e. CA4 d30 callus); bar = 750 μm. **d**. CA4 d90 callus with poorly-differentiated green structures (arrow); bar = 3 mm. **e**. CA4 d120 callus exhibiting poorly-differentiated green structures (black arrows); necrotic area can be seen (white arrow); bar = 2.5 mm. **f**. Protocorm - like body (PLB) in early stage of development with emerging shoot primordia (arrows) in 30-day-old embryogenic/organogenic callus on A10 medium (i.e. CA10 d30 callus)**; **bar = 1 mm. **g**. CA10 d60 callus showing PLBs formed from shoot primordia; bar = 350 μm. **h**. CA10 d90 callusexhibiting cluster of PLBs and rooting; bar = 3.5 mm. **i**. CA10 d120 callusexhibiting PLB - derived plantlets; bar = 8.5 mm. **j**. Rooted plantlet on A5 medium 15 days after subculture; bar = 6.5 mm.

*In vitro *germination appeared after three months in culture with highest percentages between the fourth and sixth months. However, a weak germination percentage has been observed with the formation of a massive structure originating from each seed embryo. This structure lengthened and subsequently developed into protocorm - like structure (PLS) showing an apical meristem and root primordium covered with rhizoids (Figure 1a). The mean number of PLS germination per Petri dish were scored at 8%. These PLS developed calli after 2-4 weeks of culture on the callus induction medium (Figure 1b) which were transferred after one month on the callus maintenance medium. Subculture to fresh medium was done at 21-day intervals for a period of six months. After this period, the calli were transferred to a shoot differentiation and protocorm - like body (PLB) multiplication and elongation medium (A10 medium) with A4 medium as control. These calli on A4 medium developed very slowly into few chlorophyllous regions between 30 days and 120 days (Figure 1c-e, **arrows**). White nodular compact structures were also observed on calli CA4 d30 and could be considered as somatic embryos formation (Figure 1c, **dashed arrow**). Nevertheless, these nodular compact structures did not become chlorophyllous. By contrast, calli cultured on medium A10 became green at the tips after 30 days. These chlorophyllic tips could be considered as shoot primordial in the form of protocorm - like body (PLB) formation which originated from the surface of callus and were often slightly translucent (Figure 1f, **arrows**). When callus was more organized, the number and morphology of the PLB increased resulting in well-developed shoots clearly visible on CA10 d60 calli (Figure 1g, **arrows**). One or two roots were perceived on these calli beyond 60 days of culture (Figure 1h). Multiplication, differentiation and elongation of PLBs were observed on CA10 d120 (Figure 1i). Three-cm size well developed PLBs were excised and transferred onto the rooting medium (A5 medium) (Figure 1j) whereby roots were formed after two to three weeks.

### Histological verification of shoot organogenesis

**Figure 2:**
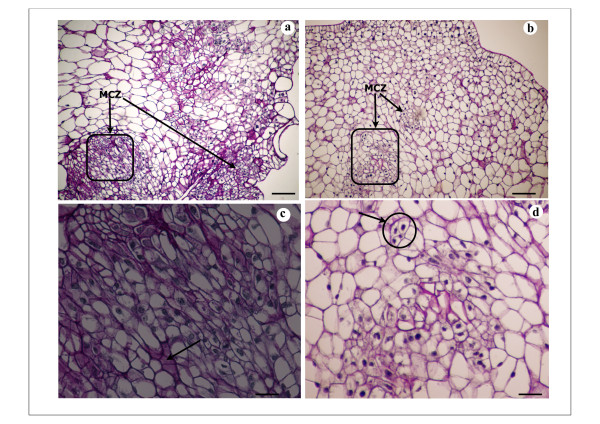
**Histological analysis of embryogenic/organogenic callus of *V. planifolia *on A4 medium and A10 medium 15 days after subculture**. Four-micrometer-thick sections of callus were stained with Periodic acid and Schiff' reagent. **a-b**. Fifteen-day-old calli on A4 medium (a) and A10 medium (b) respectively CA4 d15 and CA10 d15. Calli inner region containing both small meristematic cells with highly-stained nucleus in mitotic cells zone (MCZ) and vacuolated large cells; arrow indicate multiple MCZ; bar = 100 μm. **c-d**. Higher magnification of the MCZ in CA4 d15 callus: **c**, and in CA10 d15 callus: **d**; arrow indicate accumulation of soluble carbohydrate (sucrose and/or glucose); in **d **arrow indicates 4 thick-walled proembryonic cells; bar = 50 μm.

**Figure 3:**
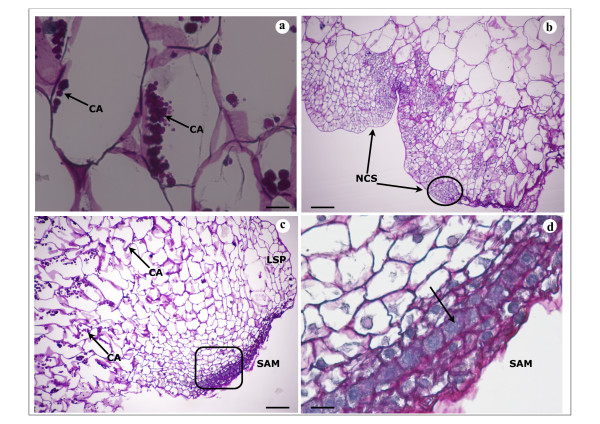
**Histological analysis of embryogenic/organogenic callus of *V. planifolia *on A4 medium and A10 medium 20 days after subculture**. Four-micrometer-thick sections of callus were stained with Periodic acid and Schiff' reagent. **a**. Staining for starch revealed cell amyloplasts (CA) in vacuolated large cells of CA10 d20 callus inner region; bar = 20 μm. The histological section from CA4 d20 showed the same figure. **b**. Nodular compact structure (NCS) containing small mitotic cell in the peripheral region of CA4 d20 callus and indicating PLB early formation; bar = 100 μm. **c**. Apex shoot differenciation in peripheral region of CA10 d20 callus: development of shoot apical meristem (SAM) and leaf sheath primodium (LSP). Cell amyloplasts (CA) near the site of shoot formation can be seen in large vacuolated cells; bar = 100 μm. **d**. Higher magnification of shoot apical meristem (SAM) in CA10 d20: cell layers in tunica development are distinguished (arrow); bar = 25 μm.

**Figure 4:**
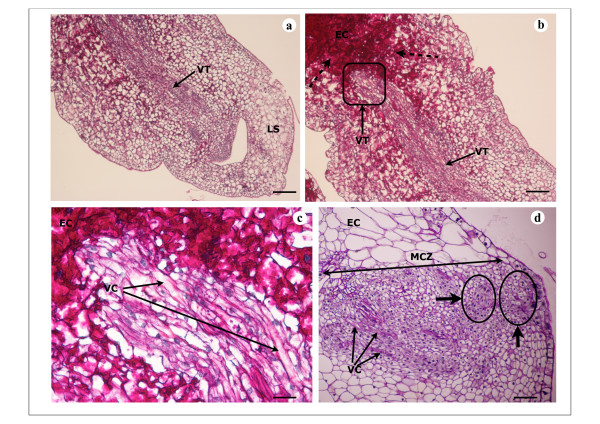
**Histological analysis of embryogenic/organogenic callus of *V. planifolia *on A4 medium and A10 medium 30 days after subculture**. Four-micrometer-thick sections of callus were stained with Periodic acid and Schiff' reagent. **a**. Demarcated and well-differentiated shoot or well-developed PLB in CA10 d30 callus. Vascular tissue (VT) and leaf sheath (LS) can be noticed; bar = 150 μm. **b**. Strong accumulation of polysaccharides (dashed arrows) in embryogenic callus (EC), particulary in posterior zone of differentiated shoot; bar = 150 μm. **c**. Higher magnification of the vascular tissue: lengthened vascular cell (VC) connected to well-developed PLB and embryogenic callus (EC) tissue that contains heavy accumulation of polysaccharides (starch); bar = 30 μm. **d**. Intense mitotic cells zone (MCZ) with vascular cells in CA4 d30; dense meristematic cells or proembryonic cells are also observed and formed PLB. No starch accumulation can be detected in embryogenic callus (EC); bar = 100 μm.

*In vitro *plant regeneration was studied and analyzed 15, 20 and 30 days after subculture of calli on either A4 medium (CA4 calli) or on A10 medium (CA10 calli). Thus, 15 days after subculture, global view showed few differences between CA4 d15 and CA10 d15 calli (Figure 2a, b). Multiple original zones with small cells with well-defined and large nuclei (Figure 2a, b, c, d) were noticed on these two types of calli and were proembryonic cells indicating the embryogenic nature of calli CA4 d15 and CA10 d15 with highly regenerative efficiency. Thus, Figure 2d showed four proembryonic cells surrounded by a thick wall that formed a young proembryon and may cause the formation of PLB in CA10 d15 calli (Figure 2d,*** arrow***). The cellular structure of these two types of calli was heterogeneous with bigger vacuolated cells in the cortex region (Figure 2a, b). The pink colour of some large cells in CA4 d15 and CA10 d15 calli after PAS staining indicates that these cells probably accumulate soluble carbohydrates such as sucrose and/or glucose (Figure 2c*** arrow***). Starch grains were visible after 20 days in the large cells of the cortex region of these two types of calli (Figure 3a, ***arrow***). Meristematic cells forming nodular compact structures were also detected on the peripheral region of calli CA4 d20 indicating the first step of pro-embryos development and perhaps PLB formation (Figure 3b, ***arrows***). However, at any time, no differentiated or well-developed PLB were noticed from these compact structures. Apex shoot initiation was perceived in calli CA10 d20 (Figure 3c). Starch grains were seen to accumulate in the subapical regions of these shoots (Figure 3c, ***arrows*) **whereas layers of small cells with central big nuclei and dense cytoplasm were distinguished in the apical regions. Those cell layers could be at the origin of the development of tunica what could be the beginning of PLB organogenesis in calli CA10 d20 (Figure 3d, ***arrow***). Shoot differentiation was observed in calli CA10 d30 (Figure 4a). Thus, differentiated shoots or PLB at early stage of development were characterized by a very noticeable leafsheath and made up of vascularized tissue composed of elongated cells (Figure 4a, c, ***arrows***). An important accumulation of polysaccharides probably starch especially at the base of newly differentiated PLBs was noticed in embryogenic callus (Figure 4b, **dashed *arrows***). This type of cellular differentiation was neither observed in the borders nor in the cortex regions of calli CA4 d30 which had poorly developed cellular structures recognized by their elementary vascular system (Figure 4d, **thin *arrow***). In these two regions, original stage of PLB formation from multi proembryonic cells was observed in mitotic cells zone (MCZ) (Figure 4d, **thick *arrow***). CA4 d30 calli were embryogenic calli with potential of organogenis like embryogenic/organogenic calli.

The nearby presence of starch could be an important factor in organogenesis of PLB in CA10 d20 and CA10 d30 calli. These observations confirm that Orchid seeds cannot reach the seedling stage without an external supply of carbohydrates which is provided in nature by fungi mycorrhizae.

### Proteomic analysis

**Figure 5:**
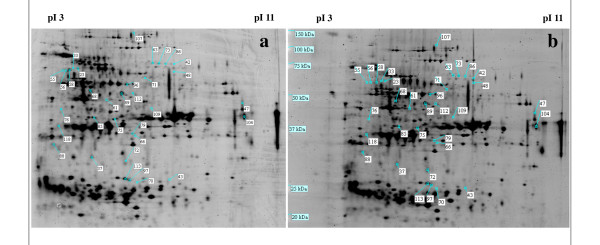
**Two-dimensional gel electrophoresis of 15-day-old *V. planifolia *organogenic callus proteome**. The images represent the expressed proteins profiles obtained at day 15 from organogenic calli on A4 medium (**gel a**) and A10 medium (**gel b**), respectively CA4 d15 and CA10 d15. The spots showing significantly differential expression are labeled.

**Figure 6:**
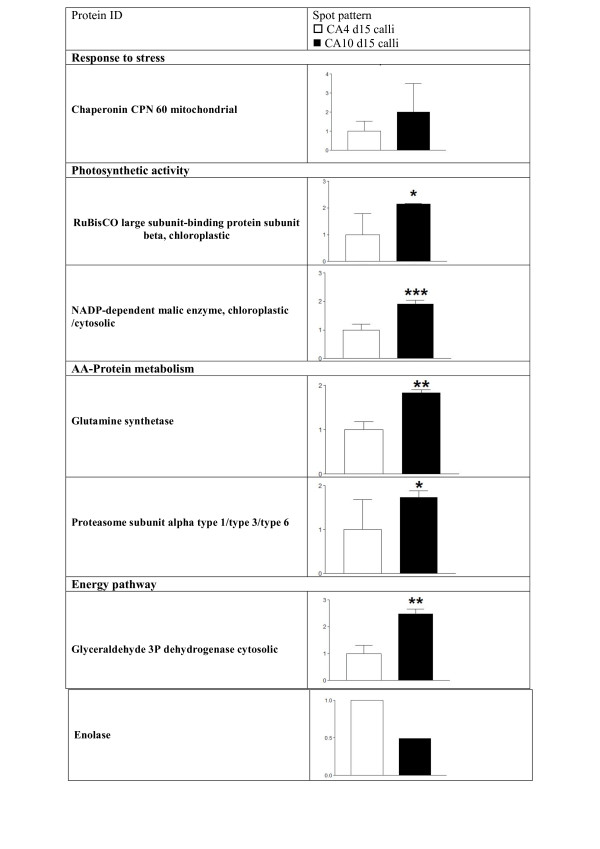
**Relative abundance of differentially accumulated major proteins upregulated and identified in CA4 d15 and CA10 d15 calli**. Additional informations on protein identification are reported in Additional file 2. The asterisks indicate significant changes in protein abundance in CA10 d15 calli as compared to CA4 d15 calli (*P < 0.05; ** *P *< 0.01; ***P < 0.001).

**Figure 7:**
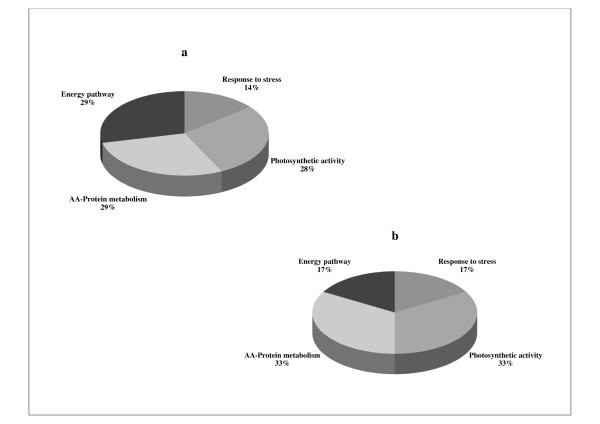
**An overview of the function classification of proteins in 15-day-old *V. planifolia *organogenic callus culture**. **a: **the proteins upregulated and identified in CA4 d15 and CA10 d15 calli (7 proteins were identified); **b: **the proteins only upregulated in CA10 d15 calli (6 proteins were identified). Each area in **a **or **b **represents the relative percentage of number of proteins identified in each category by the total number of proteins identified.

The regeneration of plantlets via indirect shoot organogenesis on A10 medium was confirmed using histological techniques. No bipolar structures (unique feature of somatic embryos) could be identified. Vascular connections were also detected between developing shoots and callus tissue. This is another distinct feature of shoot organogenesis. Based on these results, shoot differentiation had probably started between d15 and d20 after subculture of organogenic calli onto A10 medium. Thus, the earlier changes in protein expression profiles were analyzed by comparing the 2-DE proteome of CA10 d15 and CA4 d15 calli at the stage where no differentiating part was visible in CA10 d15 calli morphology (Figure 5). The two 2-DE images had a similar pattern of protein spots distribution. The majority of them (70% of the total spots) did not show any change. However, 33 protein spots with significantly different (*P *< 0.05) expression levels were retained on calli cultured in two conditions (A4 medium and A10 medium; Additional file 1). By using MALDI-TOF-TOF-MS analysis and MS/MS database, 15 differentially expressed protein spots were more reliable identified. Additional file 2 provides the Swiss-Prot accession numbers corresponding to protein spots shown in Figure 5, the putative names of proteins, the plant organism from which the protein has been identified, the number of amino acid matched, the confidence percentage (unused score), the percentage of identification, the sequence of identified peptides and the values for experimental and theoretical molecular mass. Unfortunately, 7 protein spots (43, 70, 72, 85, 86, 96 and 104) were identified with only 1 peptide sequence matched and 2 protein spots (61 and 89) were identified with other organisms. No homology with known peptide sequence in available protein databases was perceived for 9 protein spots (47, 66, 71, 76, 79, 107, 109, 112 and 118). These 15 protein spots which were clearly identified represent 7 different proteins according to their description. These 7 proteins have been classified into 4 categories based on their potential functions (Figure 6). The majority of these proteins are those involved in amino acid-protein metabolism (29%), energy pathways metabolism (29%) and photosynthetic activity (28%). The other proteins are those engaged in stress response (14%). This distribution changes if we consider only proteins upregulated in CA10 d15 calli which represent 6 out of the 7 proteins identified both on CA4 d15 and CA10 d15 calli. These 6 proteins could also be classified with the relative percentage such that the majority was those involved in photosynthetic activity (33%) and amino acid-protein metabolism (33%) (Figure 7b). However, for this novel distribution, the relative percentage of stress induced proteins increases to 17%. These proteins belong to a category of widespread proteins, chaperonin CPN 60.

### Metabolic profiling

#### Metabolic elucidation using one and two dimensional NMR spectroscopy

**Figure 8:**
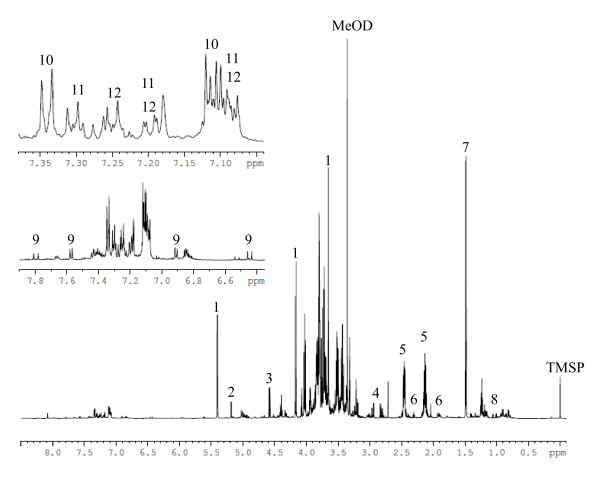
**Typical ^1^H NMR spectra (methanol- *d*_4_-KH_2_PO_4 _in D_2_O, pH 6.0 extract) of CA4 d15 callus in the range of δ 0 - 8.5, 6.5 - 7.9 and 7.0 - 7.4**. Assignments: 1, sucrose; 2, α-glucose; 3, β-glucose; 4, asparagine; 5, glutamine; 6, δ-aminobutyric acid; 7, alanine; 8, valine; 9, *p*-coumaric acid; 10, *p-*hydroxybenzyl alcohol glucoside; 11, bis [4-(β-D-glucopyranosyloxy)-benzyl]-2-isopropyltartrate (glucoside A); 12, bis [4-(β-D-glucopyranosyloxy)-benzyl]-2-(2- butyl)tartrate (glucoside B).

One- (1D) and two-dimensional (2D) NMR spectroscopy techniques are reliable methods for the analysis of a broad range of compound allowing the identification of compounds such as amino acids, carbohydrates, organic acids and phenolic compounds. 2D NMR spectroscopy, such as J-resolved, COSY (correlation spectroscopy) and HMBC (heteronuclear multiple bond correlation), was applied to identify metabolites from the congested ^1^H NMR signals. Thus, high-resolution NMR analysis has the potential to reveal the metabolic effect of enzymatic expression changes observed at proteomic level. Figure 8 shows a ^1^H NMR spectrum of CA4 d15 calli metabolome. Signals at δ 5.40 (d, *J *= 3.8 Hz), δ 5.20 (d, *J *= 3.8 Hz), δ 4.59 (d, *J *= 7.9 Hz), δ 4.17 (d, *J *= 8.6 Hz) were assigned to the anomeric protons of glucose moiety of sucrose, α-glucose, β-glucose and the fructose moiety of sucrose, respectively. Amino acids were identified at δ 2.82 (dd, *J *= 17.0, 8.2 Hz) and δ 2.95 (dd, *J *= 17.0, 4.0 Hz) as asparagine, at δ 2.13 (m) and d 2.46 (m) as glutamine, at δ 1.48 (d, *J *= 7.2 Hz) as alanine, at δ 1.01 (d, *J *= 6.8 Hz) and δ 1.06 (d, *J *= 6.8 Hz) as valine. In addition to these compounds, δ-aminobutyric acid (GABA) was identified at d 3.01 (t, *J *= 7.5 Hz), δ 2.30 (t, *J *= 7.5 Hz) and δ 1.90 (q, *J *= 7.5 Hz) (Figure 8).

In the aromatic region (δ 5.7-9.0), signals at d 7.80 (d, *J *= 16.0 Hz), δ 7.58 (d, *J *= 9.8 Hz), δ 6.91 (d, *J *= 9.8 Hz) and δ 6.45 (d, *J *= 16.0 Hz) were assigned to *p*-coumaric acid; signals at δ 7.34 (d, *J *= 9.0 Hz), δ 7.11 (d, *J *= 9.0 Hz), δ 4.57 (s), δ 5.02 (d, *J *= 7.9 Hz) were assigned to *p-*hydroxybenzyl alcohol glucoside; signals at δ 2.20 (m), δ 0.92 (d, *J *= 7.0 Hz), δ 0.86 (d, *J *= 7.0 Hz) were assigned to bis [4-(β-D-glucopyranosyloxy)-benzyl]-2-isopropyltartrate (glucoside A) and signals at δ 1.90 (m), δ 1.35 (m), 1.10 (m), δ 0.84 (d, *J *= 7.0 Hz), δ 0.77 (t, *J *= 15.0 Hz) were assigned to bis [4-(β-D-glucopyranosyloxy)-benzyl]-2-(2- butyl)tartrate (glucoside B) [23]. Table 1 summarizes all compounds identified in ^1^H NMR spectra with the chemical shifts and the coupling constants of the signals. No qualitative differences were found between CA4 d15 and CA10 d15 calli metabolic profile (Additional file 3).

**Table 1:** ^1^H Chemical Shifts (δ), Coupling Constants (Hz) of CA4 d15 and CA10 d15 calli metabolites identified by references and using 1D and 2D NMR spectra (methanol-*d*_4_-KH_2_PO_4 _in D_2 _O, pH 6.0).

No	Compound	Chemical shifts and coupling constants
1	Sucrose	δ 5.40 (H-1, d, *J *= 3.8 Hz), δ 4.17 (H-1', d, *J *= 8.6 Hz)
2	α-Glucose	δ 5.20 (H-1, d, *J *= 3.8 Hz)
3	β-Glucose	δ 4.59 (H-1, d, *J *= 7.9 Hz)
4	Asparagine	δ 2.82 (H-3a, dd, *J *= 17.0, 8.2 Hz), δ 2.95 (H-3b, dd, *J *= 17.0, 4.0 Hz)
5	Glutamine	δ 2.13 (H-4, m), δ 2.46 (H-3, m)
6	γ-aminobutyric acid (GABA)	δ 3.01 (H-4, t, *J *= 7.5 Hz), δ 2.30 (H-2, t, *J *= 7.5 Hz), δ 1.90 (H-3, q, *J *= 7.5 Hz)
7	Alanine	δ 1.48 (H-3, d, *J *= 7.2 Hz)
8	Valine	δ 1.01 (H-4, d, *J *= 6.8 Hz), δ 1.06 (H-5, d, *J *= 6.8 Hz)
9	*p-*coumaric acid	δ 7.80 (H-7, d, *J *= 16.0 Hz), δ 7.58 (H-3, H-5, d, *J *= 9.8 Hz), δ 6.91 (H-2, H-6, d, *J *= 9.8 Hz), δ 6.45 (H-8, d, *J *= 16.0 Hz)
10	*p-*hydroxybenzyl alcohol glucoside	δ 7.34 (H-3, H-5, d, *J *= 9.0 Hz), δ 7.11 (H-2, H-6, d, *J *= 9.0 Hz), δ 4.57 (H-7, s), δ 5.02 (H-1', d, *J *= 7.9 Hz)
11	bis [4-(β-D-glucopyranosyloxy)-benzyl]-2-isopropyltartrate (glucoside A)	δ 2.20 (m), δ 0.92 (d, *J *= 7.0 Hz), δ 0.86 (d, *J *= 7.0 Hz)
12	bis [4-(β-D-glucopyranosyloxy)-benzyl]-2-(2- butyl)tartrate (glucoside B)	δ 1.90 (m), δ 1.35 (m), 1.10 (m), δ 0.84 (d, *J *= 7.0 Hz), δ 0.77 (t, *J *= 15.0 Hz)

**Figure 9:**
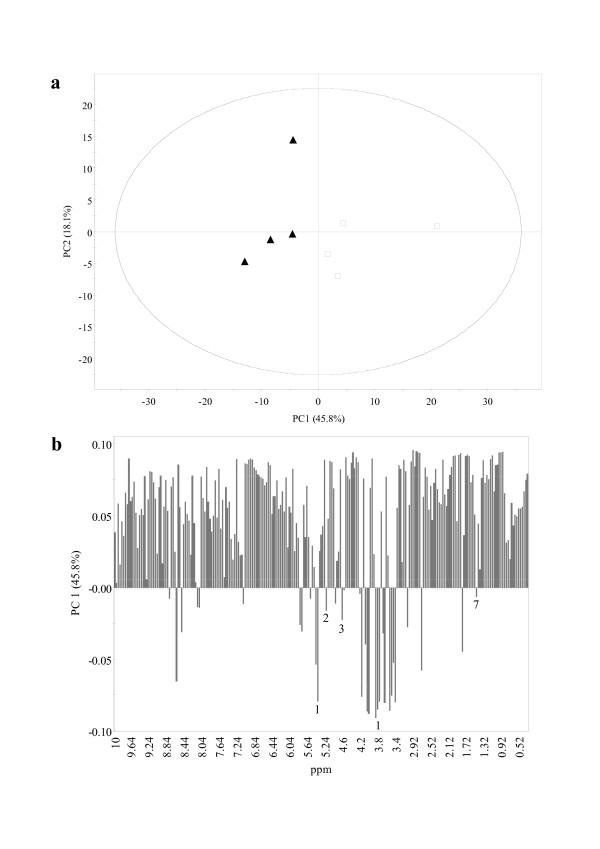
**a: Score plot (PC1 vs. PC2) and b: loading column plot of PCA results obtained from ^1 ^H NMR spectra of CA4 d15 (▲) and CA10 d15 (□) calli**. Assignments in **b: **1, sucrose; 2, α-glucose; 3, β-glucose; 7, alanine.

Principal component analysis is an unsupervised clustering method requiring no knowledge of the data set and acts to reduce the dimensionality of multivariate data analysis while preserving most of the variance within it [24]. The two first principal components (PC1 and PC2) explained 63.9% of the variation in the entire dataset (Figure 9a). Although PC1 discriminates both CA4 d15 and CA10 d15 calli, the intra-group variation is not negligible compared to the inter-group variation. Indeed, no qualitative differences between CA4 d15 and CA10 d15 calli were inferred from the visual inspection of the ^1^H NMR spectra. Furthermore, few quantitative differences were observed; as a result, there is not a strong separation between CA4 d15 and CA10 d15 calli in the PCA score plot (Figure 9a). Nevertheless, as shown by the loading column plot of PC1, CA4 d15 calli were characterized by high content of sucrose, glucose and alanine while the other compounds detected were in a higher amount in CA10 d15 calli (Figure 9b). Analysis of CA4 d15 and CA10 d15 calli were also performed by LC-MS spectroscopy and confirmed the identification of phenolic compounds by ion mass detection of the compounds (data not shown).

## Discussion

In this work, the induction of shoot organogenesis from protocorm callus of *V. planifolia *was initiated for the first time. Using an integrated approach combining proteomics and metabolomics, biochemical and physiological markers at the origin of callus organogenesis were explored.

### Organogenic callus culture

Alone or in combination with auxin, several effects of TDZ in Orchids tissue culture have been reported [25,26]. In this study, protocorm-like structures were initiated by culturing *V. planifolia *seeds on basal medium containing 0.5 mg l^-1 ^TDZ. Callus induction and maintenance from these PLS required the presence of IAA and TDZ. The presence of these two hormones in the induction and maintenance of callus (A4 medium) may contribute to the establishment and maintenance of the embryogenic/organogenic state of the calli. In fact, TDZ is considered to be one of the most active cytokinins for shoot induction in plant tissue culture [27] and in particular Orchids tissue culture [28,29]. In tissue culture TDZ could promote synthesis or accumulation of endogenous cytokinins [27]. TDZ could also influence endogenous levels of IAA [29,30]. In fact, calli on A4 medium containing 0.3 mg l^-1 ^TDZ and 0.5 mg l^-1 ^IAA and did not develop roots at any instance. Conversely, slightly differentiated chlorophyllic structures were clearly perceived after three months culture on the same medium (Figure 1e). Even if no endogenous plant hormone analysis were made in this work, these results lead us to think about endogenous ratios of auxin/cytokinin in calli cultured on A4 medium. Endogenous cytokinins might not be at an adequate concentration to induce shoots differentiation.

### Shoot differentiation

Exogenous TDZ (0.3 mg l^-1^) supplied on A4 medium was probably not suitable to induce shoot differentiation or PLB organogenesis. This was possibly the case for calli CA4. Conversely, these embryogenic/organogenic calli differentiate into shoots between 15 to 20 days after subculture on A10 medium which contains NAA (0.5 mg l^-1^) and 30 days after subculture, PLBs were well-developed on callus surface. Shoot differentiation was probably induced by the removal of TDZ and perhaps stimulated by the modulating effect of NAA on the level of endogenous cytokinins. Such modulation of NAA on cytokinin metabolism was reported by Nordstrom et al. [31]; Yin et al. [12]. The hypothesis of a high endogeneous level of cytokinins in embryogenic/organogenic calli should no be rejected. This hypothesis is supported by the additional experiments carried out in this work. As such, transfer of these six-months-maintained embryogenic/organogenic calli from A4 medium to hormone-free basal medium (A5 medium), has given rise to highly differentiated shoots similar to PLBs developed by calli CA10 although the development of these shoots was slower (Additional file 4). However, the hypothesis of a possible direct effect of TDZ should not be discarded either. Regarding our results, well-development of PLBs was stimulated in the presence of NAA. Similar results have been reported by Roy and al. [32].

### Proteomic analysis of organogenic callus differentiation process

In order to understand the biochemical and molecular events underlying this PLB organogenesis in embryogenic/organogenic callus differentiation, 2-D gel electrophoresis associated with MALDI-TOF-TOF-MS approach was used to identify proteins that were differentially regulated, despite the lack of information about *V. planifolia *sequenced genome. This study constitutes the first proteomic analysis of vanilla. Among the differentially expressed protein in CA10 d15 calli, amino acid-protein metabolism proteins and photosynthetic activity proteins were the top two protein categories demonstrating their important role in the early stage of vanilla callus differentiation process. The other upregulated detected proteins were from energy pathways and response to stress protein categories. The mitochondrial chaperonin CPN60, is the only one stress response protein showing a high level of expression. Chaperone proteins are overexpressed under several types of stress, including maintenance of cells during early culture or during initiation of *in vitro *plant development [13,33]. Chaperone proteins are also known for their roles in the maturation of protein complexes and facilitate the folding process of newly synthesized proteins. In our study, stimulation of mitochondrial CPN60 could also be related to the transition of cellular proliferation status to cellular differentiation status in CA10 d15 calli. Indirect support for this hypothesis is the observation that chaperone proteins were expressed at the breaker stage between tomato pericarp development and ripening [34]. So, the cell reprogramming and differentiation require the synthesis, assembling and stabilization of proteins as described by [16,18]. Therefore it is not surprising that glutamine synthetase and proteasome complex, two proteins involved in amino acid - protein metabolism are significantly upregulated in CA10 d15 calli, thus showing the same trend at early stages of callus differentiation (Figure 6). Indeed, glutamine synthetase is known to play a central role in plant nitrogen metabolism by increasing amides and amino acids synthesis required for protein synthesis [35]. On the other hand, the proteasome complex is associated with the breaking down process and degradation of denatured proteins or abnormal proteins that encountered errors in synthesis during rapid cell division [18,36].

With reference to proteins involved in energy mobilization, our results show that cytosolic glyceraldehyde 3-phosphate dehydrogenase (GAPDH) was significantly enhanced in calli CA10 (Figure 6). This glycolytic enzyme is often involved in *in vitro *organogenesis and somatic embryogenesis processes [15,37,38]. Hence, enhanced glycolysis is probably involved in cellular division and differentiation in calli CA10. Sucrose mobilization and utilization from the culture medium is probably responsible for the activity of this enzyme involved in the glycolysis pathway. Other proteins involved in carbohydrate metabolism and over expressed in calli CA10 d15, are two major enzymes involved in the Calvin's cycle (RuBisCo large subunit-binding protein subunit-beta and NADP-dependant malic enzyme. This suggest that the initiation of organogenesis in calli CA10 d15 is correlated to the setting up of photosynthesis system as another energy source as reported by Yin et al. [11].

### Metabolomic analysis of organogenic callus differentiation process

Metabolite analysis was also conducted on the two calli samples (CA4 d15 calli and CA10 d15 calli) for four independent repeats of the biological experiment. For each sample the first three metabolite extractions from the first three independent repeats were done from the same calli used for protein extraction.

**Figure 10:**
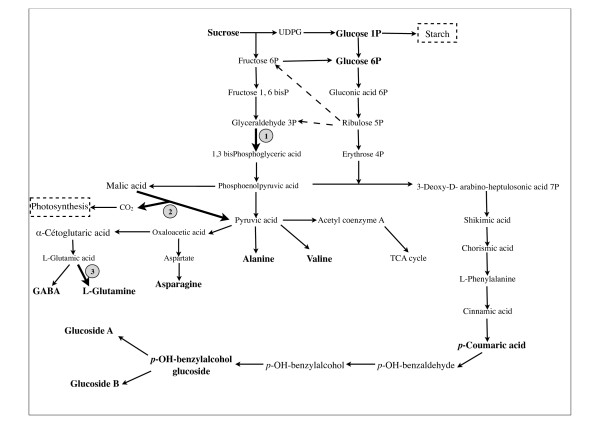
**Metabolic pathways leading to synthesis of metabolites found differentially present in CA10 d15 calli**. Metacyc database was used to elucidate metabolic networks http://metacyc.org. The enzymatic reactions that were upregulated and clearly identified by proteomic analysis are shown with full arrows 1: Glyceraldehyde-3-phosphate deshydrogenase; 2: NADP-dependent malic enzyme; 3: Glutamine synthetase). Dashed arrows represent glycolysis indirect reinforcement. Metabolite that accumulated and identified by NMR are shown in bold. For simplicity, all reactions were shown as unidirectional. The processes of starch accumulation and photosynthetic activity were induced.

In this study, sucrose, glucose and alanine contents were in a higher amount in CA4 d15. Although the amount of asparagine, glutamine, valine and phenolic compounds detected were higher in the shoot differentiation calli (CA10 d15 calli), none of these changes was statistically significant. Perhaps this is due to the small number of samples and/or the small metabolic variation between CA4 d15 and CA10 d15 calli. In addition, the calli cultures were stopped after 15 days at the stage where no visible differences between the CA4 and CA10 calli were observed; so CA10 calli were just at an early stage of the shoot differentiation and possible related metabolomic changes. This could explain the small difference observed between the two types of calli. Nevertheless, our results suggest that there is an increase in secondary metabolites (i.e. phenolic compounds) and a decrease in sugars (i.e. sucrose and glucose) in the early stages of shoot differentiation (Figure 10). Indeed, phenolic compounds are formed from the glycolysis via the shikimic acid pathway [37-39]. These metabolomic results are in accordance with those obtained from the proteomic analysis where enzymes from the glycolysis pathway were more expressed in the CA10 d15 calli as compared to the CA4 d15 calli. The same is observed for amino acids and proteome changes such as the glutamine synthetase upregulation in CA10 d15 calli. Glutamine synthetase plays a major role in plant nitrogen metabolism [35]. Thus, among the amino acids involved in plant developmental process, GABA, asparagine and glutamine seem to accumulate in CA10 d15 calli as also observed by Bender et al. [40], Murch et al. [41], Dowlatabali et al. [10].

Interestingly, the phenolic compounds identified in the callus from *V. planifolia *are known as intermediates in the vanillin biosynthesis. Indeed, *p-*hydroxybenzylalcohol glucoside, bis [4-(β-D-glucopyranosyloxy)-benzyl]-2-isopropyltartrate (glucoside A); 12, bis [4-(β-D-glucopyranosyloxy)-benzyl]-2-(2- butyl)tartrate (glucoside B) were detected previously in *V. planifolia *green beans [23,42]. Our results highlight the importance of the *p-*coumaric acid as precursor in the vanillin biosynthetic pathway [43,44]. Furthermore, glucoside A and B were never reported previously in *V. planifolia *cell or tissue culture. For this study, calli were obtained from protocorm of *V. planifolia *seeds. Inside the green pods, the accumulation area of glucovanillin was shown to be very close to the seeds [45]. In these conditions, callus obtained from protocorm could be a material of choice for the study of the vanillin biosynthetic pathway. In our experiments, *p-*hydroxybenzylalcohol and glucosides A and B particularly are present in calli at early stage of shoot differentiation in CA10 calli. This could mean that those compounds in *V. planifolia *are accumulated in the younger or photosynthetic tissues (i.e. embryos or leaves) and then transported and converted to glucovanillin in the specialized tissues during green pod development as it has been observed previously [23,42].

## Conclusion

In this study for the first time an integrated approach, incorporating two highly complementary techniques, proteomics and metabolomics analysis, was used in Orchidaceae plant's developmental process research. We investigated the effects of exogenous plant hormones (removal of TDZ and addition of NAA) on the biochemical mechanisms that release shoot formation at early stage of PLB organogenesis during *V. planifolia *totipotent callus differentiation. The cellular reprogramming in this morphogenesis process suggested an early stimulation of several metabolic pathways including mobilization of sucrose, glycolysis and phenolic compound synthesis, amino acids and protein synthesis and protein stabilization among others to assemble the photosynthetic machinery in the cells. Our next goal will be the integration of endogenous hormone profiles of totipotent callus and PLBs elongated with proteomic and metabolomic information to obtain a large view of plant's development process.

Our results show also that already at a very early stage of plant development coumaric acid and glucoside A and B are produced. As the compounds are precursors for vanillin, it might point to the possibility that metabolomics could be used at an early plant stage to determine the plants potential to produce vanillin. Further studies should be of interest.

## Methods

### Plant material

Seven-months-old green pods of *V. planifolia *(flavour elite, Accession number 13B3) were collected and immediately disinfected in 50% hydrogen peroxide solution. This pre-sterilization treatment was followed by complete sterilization in the laminar airflow cabinet using a protocol established by Kodja et al. [46]. The seeds were then removed from the pod, detached and 200-300 seeds were spread out in 9 cm Petri dishes. These seeds were then cultured in the dark on a germination medium composed with basal medium (BM) containing TDZ (Table 2; see additional file 5 for preliminary experiments) for the production of protocorm like structures (PLS). The basal medium was composed of Murashige and Skoog [47] macro and microelements, Morel and Wetmore [48] vitamins and 30 g l^-1 ^sucrose. BM was supplemented with different growth regulators for callus induction from PLS, callus maintenance, differentiation, multiplication and elongation of shoots from calli and rooting of shoots (Table 2). Maintenance of calli was carried out by subculture on A4 medium at 21-day intervals for a six-month period prior to morphological, cytological and biochemical studies. The calli were transferred to A10 medium to induce differentiation, multiplication and elongation of shoots after this stabilization period. Calli on A10 medium, (CA10) were cultivated during 4 months without subculture. Morphological (Additional file 6), cytological and/or biochemical studies were effected on day 15 (d15), day 20 (d20), day 30 (d30), day 60 (d60), day 75 (d75), day 90 (d90) and day 120 (d120). In parallel and as control, calli were cultured on A4 medium (CA4) from day 15 until day 120 for the same morphological, cytological and/or biochemical analyses to check if the expected shoot formation had been induced onto A4 medium. Culture conditions from calli induction from PLS to rooting of shoots consisted of 16 h photoperiod maintained by white cooled fluorescent tubes (NL36w/830) providing a light intensity of 55 μmol m^2 ^s^-1 ^in a growth room set at 25 ± 1°C. All culture media were autoclaved at 120°C after adjusting the pH to 5.8 by using 1 M KOH.

**Table 2:** Procedure of plantlet regeneration from callus derived protocorm of *V. planifolia*.

	Stage of plantlet micropropagation				
	
Plant growth regulators in basal medium (BM)	PLS germination	Embryogenic callus induction on callus induction medium	Embryogenic/organogenic callus culture on callus maintenance medium (A4 medium)	Shoot differentiation, PLB multiplication and elongation on A10 medium	PLB - derived plantlet rooting on A5 medium
Thidiazuron (TDZ)	0.5 mg l^-1^	0.5 mg l^-1^	0.3 mg l^-1^	0	0
Indole-3-acetic acid (IAA)	0	0.5 mg l^-1^	0.5 mg l^-1^	0	0
α-Naphthalene acetic acid (NAA)	0	0	0	0.5 mg l^-1^	0
Phytagel	7.5 g l^-1^	7.5 g l^-1^	7.5 g l^-1^	7.5 g l^-1^	7.5 g l^-1^
Other features					
Container	Petri disk	Petri disk	tube	Flask	tube
Medium volume	20 ml	20 ml	20 ml	100 ml	20 ml
Subcultures	No	No	Every 21 days	No	No
Culture time	4 - 6 months	2 months	6 months	4 months	2 months

In the same way as the experiment of shoot differentiation and multiplication on A10 medium with CA10 calli, organogenic calli were cultured on A4 medium (CA4 calli) as control. A4, A5 and A10 media were chosen from results of preliminary experiments (see Additional file 5).

### Histology and histochemistry

To verify the occurrence of indirect shoot organogenesis, CA10 samples were collected 15, 20 and 30 days after transfer of calli onto A10 medium and CA10 d15, CA10 d20 and CA10 d30 calli were studied. Calli were fixed in formol 4% (Sigma-Aldrich), for 24 h at 4°C, transferred to automat system (Leica ASP 300) and then dehydrated under vacuum through a graded ethanol series (90%, 95, 100, 100, 100, 100%) for 1 h each time; three times in methylcyclohexane (Sigma-Aldrich) for 1 h each time and embedded in paraffin (melting point: 58°C) after two immersions of 1 h for each. Control samples (CA4 d15, CA4 d20, CA4 d30) removed from A4 medium were also fixed as described above. Serials sections of 4 μm thickness were obtained with rotary ultramicrotome (Leica RM 2125 RT, Germany). For starch, other carbohydrates and nuclei detection, callus sections were stained in 0.5% (v/v) Periodic acid (Sigma-Aldrich) for 10 min and 20 min in Schiff' reagent. Callus sections were rinsed with distilled water and dehydrated in 100% ethanol followed by quick immersion in toluene (Sigma-Aldrich). Sections were stretched and mounted on glass slides (SuperFrost^®^, Menzel-Glaser) and were examined under Nikon's Eclipse TE 2000-U microscope. Photographs were taken using a digital camera of the same microscope.

### Proteomic analysis

#### Protein extraction

The proteomic profile CA10 d15 and CA4 d15 calli were studied. 700 μl of extraction buffer [49] was added to about 300 mg of calli in an Eppendorf tube and ground for 5 seconds (20 vibrations.sec^-1^) in a ball mill (MM301, Retsch, Belgium). The ground sample was centrifuged at 12 000 rpm for 10 min at 4°C. The extracted proteins were purified by using the 2D Clean-Up kit (GE-Healthcare, UK). 150 μl samples were used to recover proteins in 30 μl urea buffer, 7 M urea, 2 M thiourea, 4% CHAPS, 2% DTT, 2% IPG Buffer. Proteins were quantified using the FluoroProfile^® ^Protein Quantification Kit (Sigma-Aldrich, USA). Three independent protein extractions from three independent repeats of biological experiment were done for each sample of CA4 and CA10. Each extraction was analyzed by two gel replicates.

#### 2-DE (IEF/SDS- PAGE)

IEF was carried out on immobiline dry strip gels (18 cm) with a Non linear pH gradient (pH 3-11) [50]. The strips were rehydrated for 15 h at room temperature in 340 μl of rehydrating solution (DeStreak Rehydration Solution, GE Healthcare) to which 15 μl ampholyte solution was added (IPG Buffer 3-11NL, GE Healthcare). Isoelectrical focusing was done on the Ettan IPGphor 3 IEF System (Ge Healthcare). 190 μg protein of each sample was loaded on the strip and IEF was performed on the Ettan IPGphor 3 IEF System (GE-Healthcare) in 4 steps for 9 h at 20°C applying the following program: the first step occurred at 500 V during 1 h, followed by a linear increase from 500 V to 1000 V over 4 h, 1000 V to 8000 V over 3 h and finally held 8000 V for 1 h in the final step. The second phase (SDS-PAGE) was carried out on 12.5% polyacrylamide gel. The proteins on the strips were reduced in an equilibration buffer composed of 6 M urea, 50 mM Tris-HCl pH = 8.8, 30% glycerol, 2% SDS, 0.02% bromophenol blue and added with 1% W/V DTT for 10 min. Then the proteins were alkylated for 10 min in the same buffer added with 2.5% W/V iodoacetamide. The strips were loaded on the gel and migration occurred at 25°C for 5 h at 17 W/gel.

#### Stain and Analysis of 2D gels

Gels were stained in SyproRuby protein gel stain luminescent solution (Invitrogen, Carlsbad, CA, USA) in accordance with supplier's instructions. Protein fixation was performed overnight in 50% methanol, 7% acetic acid with gentle agitation at room temperature. Then electrophoresis gels were stained with Sypro Ruby solution under similar conditions. Finally, they were destained in 10% methanol, 7% acetic acid for 1 h. Thereafter fluorescent image of each gel was acquired using an Ettan Dige imager (GE Healthcare, Piscataway, NJ, USA). Image of gels were processed using Progenesis Samespots software (Nonlinear Dynamics, Newcastle, UK) which allows images alignment, spot detection and matching, measure of volume of each protein spot and spots quantitative comparison on all gels. Protein abundance was expressed as relative volume (% vol). Statistical analysis of the data allows protein detection with significant expression difference between control group and experimental group ANOVA test (*P *< 0.05) and fold (> 1.5) based. These spots were picked using Ettan Spot Picker (GE Healthcare, Piscataway, NJ, USA) for further analysis in mass spectrometry.

#### Protein in gel digestion

Tryptic digestion of interesting spots were performed using the ProteoExtract All-in-One Trypsin Digestion Kit (Merck Chemicals Ltd., Nottingham, UK) in accordance with supplier's instructions, with overnight incubation of samples at 37°C in the presence of trypsin final concentration of 4 ng μl^-1 ^as the final step. The tryptic fragments were desalted using μC18 ZipTips (Millipore, Billerica, USA) in three steps. Firstly, the column was activated three times with 3 μl acetonitrile 100% and equilibrated three times with TFA 0.1%. Then samples were loaded onto the column, desalted with TFA 0.1% and eluted directly onto MALDI plate with 5 mg ml^-1 ^a-cyano-4-hydroxycinnamic acid (CHCA)(LaserBio. Labs, France) matrix dissolved in 50% TFA, 50% ACN.

#### Protein identification

MS and MS/MS analyses of spotted peptide samples were performed using the 4800 MALDI-TOF/TOF Analyser (Applied Biosystems, Foster city, CA, USA). After screening the sample position in MS-positive reflector mode using 1500 laser shots, the fragmentation of automatically-selected precursors was performed at collision energy of 1 kV using air as collision gas (pressure of ~2 × 10^-6 ^Torr). MS spectra were acquired between *m/z *800 and 4000. For calibration a mixture of 4 peptides of known masses (Applied Biosytems kit) was used. Up to 12 of the most intense ion signals having a S/N > 12 were selected as precursors for MS/MS acquisition. Calibration was done at 1570.677. Peptide and protein identification were performed by the ProteinPilot™ Software V 2.0 (Applied Biosystems) using the Paragon algorithm. Each MS/MS spectrum was searched for all species against the Uniprot _sprot_20080123 database. The searches were run using the Fixed modification of iodoacetamide labelled cysteine parameter enabled. Other parameters such as tryptic cleavage specificity, precursor ion mass accuracy and fragment ion mass accuracy are MALDI 4800 built-in functions of Protein Pilot software.

The ProteinPilot software calculates a confidence percentage (the unused score) which reflects the probability that the hit is a false positive, meaning that at 95% confidence level, there is a false positive identification chance of about 5%.

### NMR Analysis

#### Extraction

Freeze-dried of totality of CA4 d15 or CA10 d15 calli material (50 mg) was transferred to a 2 ml microtube. A volume of 1.5 ml of a mixture of KH_2_PO_4 _buffer (pH 6.0) in D_2_O containing 0.05% trimethylsilylpropionic acid sodium salt (TMSP, w/w) and methanol-*d*_4 _(1:1) was added to the callus samples. The mixture was vortexed at room temperature for 1 min, ultrasonicated for 20 min, and centrifuged at 13000 rpm for 10 min. An aliquot of 0.8 ml was used for NMR analysis.

#### Measurements

^1^H NMR, 2D-J resolved, ^1^H-^1^H correlated spectroscopy (COSY), and heteronuclear multiple bonds coherence (HMBC) spectra were recorded at 25°C on a 600 MHz Bruker AV 600 spectrometer equipped with cryo-probe operating at a proton NMR frequency of 600.13 MHz. CD_3_OD was used as the internal lock. Each ^1^H NMR spectrum consisted of 128 scans requiring 10 min acquisition time with the following parameters: 0.25 Hz/point, pulse width (PW) = 30° (10.8 μsec), and relaxation delay (RD) = 1.5 sec. A presaturation sequence was used to suppress the residual H_2_O signal with low power selective irradiation at the H_2_O frequency during the recycle delay. FIDs were Fourier transformed with LB = 0.3 Hz and the spectra were zerofilled to 32 K points. The resulting spectra were manually phased and baseline corrected, and calibrated to TMSP at 0.0 ppm, using Topspin (version 2.1, Bruker). All the 2D NMR parameters were the same as in our previous reports [21].

#### Data analysis

^1^H NMR spectra were automatically reduced to ASCII files using AMIX (v.3.7, Bruker Biospin). Spectral intensities were scaled to total intensity TMSP and reduced to integrated regions of equal width (0.04 ppm) corresponding to the region of d 0.30 - d 10.02. The region of d 4.70 - d 5.00 was excluded from the analysis because of the residual signal of water as well as d 3.28 - d 3.40 for residual methanol. Principal component analysis (PCA) were performed with the SIMCA-P software (v.11.0, Umetrics, Umeå, Sweden) using Unit Variance (UV) scaling method. The *t*-tests for ^1^H-NMR signals were performed using Multi-Experiment Viewer (v. 4.0) [51].

### LC-MS procedure

#### Extraction

Freeze-dried CA4 d15 or CA10 d15 callus sample (50 mg) was transferred into a 2 ml microtube. A volume of 1.5 ml of MeOH-Water (1:1) was added to the callus samples. The mixture was vortexed at room temperature for 1 min, ultrasonicated for 20 min and centrifuged at 13000 rpm for 10 min. The supernantant was then analysed with LC-MS.

#### LC-MS Analysis

The LC system employed was an Agilent CPL/SM 1100 series (Massy, France) equipped with LC/MSD Chemstation software, degasser G1322A, binary pump G1312A, autosampler G1313A, thermostated column oven G1316A, diode array detection system G1315B to monitor at all wavelengths from 200 to 400 nm, and MSD/VL mass spectrometer with APCI source. For the column, a LiChrospher 100 RP-18 (250 × 4.6 mm i.d., s-5, 5 μm) (Merck, Darmstadt, Germany), joined with a guard column LichroCART 4-4 (Merck), was used at 35°C. Gradient elution was performed with solution A, composed of 90% water at 0.1% acetic acid (pH 3.3) and 10% methanol, and solution B, comprising 70% methanol, delivered at a flow rate of 1.0 mL/min as follows: initially 100% of solution A; for the next 15 min, 70% A; for another 30 min, 65% A; for another 20 min, 60% A; for another 5 min, 5% A; and finally 0% A for 25 min. The APCI mass spectrometer conditions were as follows: negative (or positive mode if necessary) ion mode; fragmentor voltage, 70 V; capillary voltage, 4000 V; vaporizer temperature, 350°C; corona current, 15 μA; drying gas (nitrogen) flow, 11 ml min^-1^; nebulizer pressure, 60 psig; drying gas temperature, 350°C; mode scan, 10-1000 uma. The injection volume for the extract was 10 μl. For the polyphenol analysis, a library including 100 phenolic acids, catechins, flavonoids, and simple polyphenols was first made. The library was composed of HPLC retention times and UV-DAD spectra of aglycons, and a calibration table was constructed for each compound.

The mass spectrum of each reference compound was also recorded and used to confirm identification. The internal standard used was 4-formylbenzoic acid methyl ester (360 μmol). The callus extracts were analyzed using the same HPLC system. The polyphenols were identified on the basis of their retention times, UV-DAD spectra, and APCI mass spectra and quantified according to the calibration table.

## Abbreviations

2-DE: Two - dimensional electrophoresis; BM: Basal medium; COSY: Correlated spectroscopy; GAPDH: Glyceraldehyde 3-phosphate dehydrogenase; HMBC: Heteronuclear multiple bonds coherence; IAA: Indole-3-acetic acid; MALDI-TOF-TOF-MS: Matrix-assisted laser desorption ionization-time of flight-tandem mass spectrometry; NAA: 1-Naphtalene acetic acid; NMR: Nuclear Magnetic Resonance; PAS: Periodic acid Schiff; PCA: Principal component analysis; PLS: Protocorm - like structure; PLB: Protocorm - like body; TDZ: Thidiazuron.

## Authors' contributions

TLP maintained vanilla plants culture in greenhouse, flowers pollination, callus induction, performed metabolomic data by using one and two dimensional NMR spectroscopy and multivariate analysis. PM performed protein extractions, histochemical and histological analysis. IF contributed to the interpretation of proteomic data. YHC helped in metabolic profiling and multivariate analysis and critically read the manuscript. EB helped in proteomic analysis. JGS performed the vanilla plant *in vitro *regeneration. MB performed the proteomic experiments. BP performed the LC-MS analysis. RV critically revised the manuscript. HK developed the design of all experimental protocols, performed in maintenance culture of organogenic calli and histochemical, histological and proteomic analyses and wrote with TLP the manuscript. All authors read and approved the final manuscript.

## Supplementary Material

Additional file 1 **Statistical study of protein expression between two conditions CA4 d15 calli and CA10 d15 calli**. Statistical analysis of expression of 33 protein spots upregulated in CA4 d15 and CA10 d15 calli. Click here for file

Additional file 2 **Mass spectrometry analysis of 24 proteins identified and upregulated (at least 1.5 fold) in organogenic callus of *V. planifolia***. Full list of identified organogenic callus proteins whose expression levels changed between A10 medium and A4 medium during 15 days of tissue culture. Swiss-Prot accession numbers, the putative names of proteins, the organism from which the protein has been identified, the number of amino acids matched, the confidence percentage (unused score), the percentage of identification, the sequence of identified peptides and the values for experimental and theoretical molecular mass are provided. Click here for file

Additional file 3 **^1^H NMR spectra (methanol-*d*_4_-KH_2 _PO_4 _in D_2 _O, pH 6.0 extract) of CA4 d15 and CA10 d15 calli in the range of δ -0.5 - 9.0**. Visual and comparative inspection of CA4 d15 and CA10 d15 calli spectra. Assignments: 1, sucrose; 2, α-glucose; 3, β-glucose; 4, asparagine; 5, glutamine; 6, δ-aminobutyric acid; 7, alanine; 8, valine. Click here for file

Additional file 4 **PLBs, and roots formation from embryogenic/organogenic callus after 120 days culture in A5 medium and A10 medium**. Proliferating PLBs with well-developed roots on embryogenic/organogenic callus cultured on two types of shoot regeneration medium. **a and b **Embryogenic/organogenic callus in A5 medium: BM. **c and d **Embryogenic/organogenic callus in A10 medium: BM + NAA 0.5 mg l^-1 ^(CA10 d120 callus). Bars in a and c = 7.75 mm; bars in b and d = 5.6 mm. Click here for file

Additional file 5 **Morphogenic response of embryogenic callus cultures or Protocorm-like body (PLB) - derived plantlets of *V. planifolia *after transferring on different media**. Preliminary experiments to choose the best medium at each step of the procedure of plantlet regeneration from callus derived protocorm of *V. planifolia*. Click here for file

Additional file 6 **Appearance of embryogenic/organogenic calli cultivated onto A4 medium and A10 medium**. Comparison of morphologic evolution of organogenic callus on A4 medium and A10 medium during 120 days without subculture. Click here for file
