# Supplementary material for: Shoot differentiation from protocorm callus cultures of Vanilla planifolia (Orchidaceae): proteomic and metabolic responses at early stage
Source: BMC Plant Biol. 2010 May 5;10:82. doi: 10.1186/1471-2229-10-82 (PMC3095354; doi:10.1186/1471-2229-10-82)
Supplement: Additional file 1 — Statistical study of protein expression between two conditions CA4 d15 calli and CA10 d15 calli. Statistical analysis of expression of 33 protein spots upregulated in CA4 d15 and CA10 d15 calli. [file 1471-2229-10-82-S1.DOC]

**Additional file 1. Statistical study of protein expression between two conditions (CA4 d15 calli and CA10 d15 calli).**

Only the 33 protein spots which expression is statistical different between the two conditions is shown (Anova < 0.05 and fold > 1.5). The Fold is the average normalized volumes ratio between these two conditions.

| **Spot Number** | **Anova (p)** | **Fold** | **Average Normalised Volumes** | |
| --- | --- | --- | --- | --- |
|  |  |  | **CA4 d15 calli** | **CA10 d15 calli** |
| 23 | 6,467E-04 | 3,9 | 472089,57 | 1841256,591 |
| 33 | 1,000E-03 | 3 | 232825,018 | 702729,396 |
| 37 | 2,933E-04 | 2,8 | 923080,965 | 325428,55 |
| 42 | 8,000E-03 | 2,7 | 93002,584 | 247930,683 |
| 43 | 7,726E-04 | 2,5 | 293120,676 | 737786,632 |
| 47 | 1,000E-02 | 2,4 | 678475,309 | 288108,69 |
| 48 | 4,000E-03 | 2,3 | 179107,721 | 413841,453 |
| 55 | 1,000E-03 | 2,2 | 278201,229 | 601958,027 |
| 56 | 1,000E-02 | 2,2 | 189735,629 | 409238,165 |
| 58 | 1,100E-02 | 2,1 | 1140822,655 | 2424695,263 |
| 61 | 5,000E-03 | 2,1 | 77813,679 | 161563,569 |
| 63 | 6,525E-04 | 2,1 | 331124,583 | 684147,996 |
| 66 | 9,229E-05 | 2 | 67811,615 | 137792,256 |
| 68 | 8,000E-03 | 2 | 1060050,78 | 523658,192 |
| 70 | 9,000E-03 | 2 | 38838,65 | 77342,444 |
| 71 | 1,000E-02 | 1,9 | 282019,297 | 145211,606 |
| 72 | 2,000E-03 | 1,9 | 881070,23 | 456203,298 |
| 73 | 7,000E-03 | 1,9 | 400924,176 | 770275,367 |
| 75 | 4,000E-03 | 1,9 | 2012981,964 | 3838515,749 |
| 76 | 7,000E-03 | 1,9 | 142949,949 | 271022,877 |
| 79 | 6,000E-03 | 1,9 | 335530,302 | 623389,798 |
| 85 | 3,000E-03 | 1,8 | 1394930,55 | 2473176,195 |
| 86 | 1,200E-02 | 1,8 | 234972,43 | 414634,549 |
| 88 | 7,000E-03 | 1,8 | 1054688,901 | 1846698,015 |
| 89 | 1,100E-02 | 1,7 | 3479535,819 | 6015084,842 |
| 96 | 1,615E-04 | 1,6 | 2092337,548 | 3451486,507 |
| 97 | 3,000E-03 | 1,6 | 869277,006 | 1433466,366 |
| 104 | 6,000E-03 | 1,6 | 4228159,988 | 2595755,763 |
| 107 | 7,000E-03 | 1,6 | 161263,034 | 258861,832 |
| 109 | 1,000E-03 | 1,6 | 33758,665 | 53952,156 |
| 112 | 6,000E-03 | 1,6 | 177258,68 | 277532,638 |
| 113 | 3,000E-03 | 1,6 | 1895317,195 | 2966342,064 |
| 118 | 7,000E-03 | 1,6 | 626558,47 | 975760,319 |
