# Supplementary material for: Shoot differentiation from protocorm callus cultures of Vanilla planifolia (Orchidaceae): proteomic and metabolic responses at early stage
Source: BMC Plant Biol. 2010 May 5;10:82. doi: 10.1186/1471-2229-10-82 (PMC3095354; doi:10.1186/1471-2229-10-82)
Supplement: Additional file 2 — Mass spectrometry analysis of 24 proteins identified and upregulated (at least 1.5 fold) in organogenic callus of V. planifolia. Full list of identified organogenic callus proteins whose expression levels changed between A10 medium and A4 medium during 15 days of tissue culture. Swiss-Prot accession numbers, the putative names of proteins, the organism from which the protein has been identified, the number of amino acids matched, the confidence percentage (unused score), the percentage of identification, the sequence of identified peptides and the values for experimental and theoretical molecular mass are provided. [file 1471-2229-10-82-S2.DOC]

**Additional file 2**. **Mass spectrometry analysis of 15 proteins identified and upregulated (at least 1.5 fold) in organogenic callus of *V. planifolia.***

Proteins that were upregulated in CA10 d15 calli are red. Proteins that were upregulated in CA4 d15 calli are blue. The identification of protein had to be in the top hit with, at least 2 peptide sequences matched with confidence percentage or the unused score ≥ 2 and identification success rate ≥ 40 %.

| **Spot number** | **Swiss-Prot Accession number** | **Entry Name** | **Protein name** | **Plant Organism** | **Unused Score ≥ 2** | **Number of peptides matched** | **% Conf ≥ 40%** | **Sequence** | **Prec MW** | **Prec m/z** | **Theor MW** | **Theor m/z** |
| --- | --- | --- | --- | --- | --- | --- | --- | --- | --- | --- | --- | --- |
| 23 | Q05046; Q05045 | CH62_CUCMA ; CH61_CUCMA | Chaperonin CPN60-2, mitochondrial; Chaperonin CPN60-1, mitochondrial | *Cucurbita maxima* | 3 | 2 | 99,00 | GYISPYFITNQK | 1429,7 | 1430,7 | 1429,7 | 1430,7 |
|  |  |  |  |  |  |  | 90,00 | AAVEEGIVPGGGVALLYASK | 1900,0 | 1901,0 | 1900,0 | 1901,0 |
| 33 | Q05046 | CH62_CUCMA | Chaperonin CPN60-2, mitochondrial | *Cucurbita maxima* | 10 | 5 | 99,00 | AAVEEGIVPGGGVALLYASK | 1900,0 | 1901,0 | 1900,0 | 1901,0 |
|  |  |  |  |  |  |  | 99,00 | SAIELSTSDYDKEKLQER | 2111,0 | 2112,0 | 2111,0 | 2112,0 |
|  |  |  |  |  |  |  | 99,00 | GYISPYFITNQK | 1429,7 | 1430,7 | 1429,7 | 1430,7 |
|  |  |  |  |  |  |  | 99,00 | IGVQIIQNALK | 1223,7 | 1224,8 | 1223,7 | 1224,7 |
|  |  |  |  |  |  |  | 99,00 | AGIIDPLKVIR | 1193,8 | 1194,8 | 1193,7 | 1194,8 |
|  | Q43298 | CH62_MAIZE | Chaperonin CPN60-2, mitochondrial | *Zea mays* | 2 | 4 | 99,00 | AAVEEGIVPGGGVALLYASK | 1900,0 | 1901,0 | 1900,0 | 1901,0 |
|  |  |  |  |  |  |  | 99,00 | NVVIEQSFGAPK | 1287,7 | 1288,7 | 1287,7 | 1288,7 |
|  |  |  |  |  |  |  | 99,00 | IGVQIIQNALK | 1223,7 | 1224,8 | 1223,7 | 1224,7 |
|  |  |  |  |  |  |  | 99,00 | AGIIDPLKVIR | 1193,8 | 1194,8 | 1193,7 | 1194,8 |
| 37 | Q05046 | CH62_CUCMA | Chaperonin CPN60-2, mitochondrial | *Cucurbita maxima* | 16 | 8 | 99,00 | AAVEEGIVPGGGVALLYASK | 1900,0 | 1901,0 | 1900,0 | 1901,0 |
|  |  |  |  |  |  |  | 99,00 | LLEQDNPDLGYDAAKGEYVDMIK | 2564,2 | 2565,2 | 2564,2 | 2565,2 |
|  |  |  |  |  |  |  | 99,00 | SAIELSTSDYDKEKLQER | 2111,0 | 2112,0 | 2111,0 | 2112,0 |
|  |  |  |  |  |  |  | 99,00 | GYISPYFITNQK | 1429,7 | 1430,7 | 1429,7 | 1430,7 |
|  |  |  |  |  |  |  | 99,00 | AGIIDPLKVIR | 1193,7 | 1194,8 | 1193,7 | 1194,8 |
|  |  |  |  |  |  |  | 99,00 | IGVQIIQNALK | 1223,7 | 1224,7 | 1223,7 | 1224,7 |
|  |  |  |  |  |  |  | 99,00 | IGGASEAEVGEKKDR | 1544,8 | 1545,8 | 1544,8 | 1545,8 |
|  |  |  |  |  |  |  | 99,00 | TLFNELEVVEGMKLDR | 1923,9 | 1924,9 | 1924,0 | 1925,0 |
|  | Q43298 | CH62_MAIZE | Chaperonin CPN60-2, mitochondrial | *Zea mays* | 4 | 6 | 99,00 | AAVEEGIVPGGGVALLYASK | 1900,0 | 1901,0 | 1900,0 | 1901,0 |
|  |  |  |  |  |  |  | 99,00 | NVVIEQSFGAPK | 1287,7 | 1288,7 | 1287,7 | 1288,7 |
|  |  |  |  |  |  |  | 99,00 | GISMAVDAVVTNLK | 1416,7 | 1417,7 | 1416,8 | 1417,8 |
|  |  |  |  |  |  |  | 99,00 | AGIIDPLKVIR | 1193,7 | 1194,8 | 1193,7 | 1194,8 |
|  |  |  |  |  |  |  | 99,00 | IGVQIIQNALK | 1223,7 | 1224,7 | 1223,7 | 1224,7 |
|  |  |  |  |  |  |  | 99,00 | IGGASEAEVGEKKDR | 1544,8 | 1545,8 | 1544,8 | 1545,8 |
|  | Q8L7B5 | CH60B_ARATH | Chaperonin CPN60-like 1, mitochondrial | *Arabidopsis thaliana* | 2 | 6 | 99,00 | AAVEEGIVPGGGVALLYASK | 1900,0 | 1901,0 | 1900,0 | 1901,0 |
|  |  |  |  |  |  |  | 99,00 | LLEQDNPDLGYDAAKGEYVDMIK | 2564,2 | 2565,2 | 2564,2 | 2565,2 |
|  |  |  |  |  |  |  | 99,00 | AGIIDPLKVIR | 1193,7 | 1194,8 | 1193,7 | 1194,8 |
|  |  |  |  |  |  |  | 99,00 | IGVQIIQNALK | 1223,7 | 1224,7 | 1223,7 | 1224,7 |
|  |  |  |  |  |  |  | 99,00 | QRPLLIVAEDVESDALATLILNK | 2520,4 | 2521,4 | 2520,4 | 2521,4 |
|  |  |  |  |  |  |  | 99,00 | TLFNELEVVEGMKIDR | 1923,9 | 1924,9 | 1924,0 | 1925,0 |
| 42 | P34921 | G3PC_DIACA | Glyceraldehyde-3-phosphate dehydrogenase, cytosolic | *Dianthus caryophyllus* | 7,54 | 4 | 99,00 | VPTVDVSVVDLTVR | 1497,8 | 1498,8 | 1497,8 | 1498,8 |
|  |  |  |  |  |  |  | 99,00 | AASFNIIPSSTGAAK | 1433,7 | 1434,7 | 1433,8 | 1434,8 |
|  |  |  |  |  |  |  | 99,00 | AGIALNDNFIK | 1188,6 | 1189,6 | 1188,7 | 1189,7 |
|  |  |  |  |  |  |  | 96,00 | IKIGINGFGR | 1074,6 | 1075,6 | 1074,6 | 1075,6 |
|  | P17878 | G3PC_MESCR | Glyceraldehyde-3-phosphate dehydrogenase, cytosolic | *Mesembryanthemum crystallinum* | 2 | 2 | 99,00 | GILGYTEDDLVSTDFIGDNR | 2211,0 | 2212,0 | 2211,0 | 2212,0 |
|  |  |  |  |  |  |  | 99,00 | AASFNIIPSSTGAAK | 1433,7 | 1434,7 | 1433,8 | 1434,8 |
| 48 | P34921 | G3PC_DIACA | Glyceraldehyde-3-phosphate dehydrogenase, cytosolic | *Dianthus caryophyllus* | 8,09 | 5 | 99,00 | VPTVDVSVVDLTVR | 1497,9 | 1498,9 | 1497,8 | 1498,8 |
|  |  |  |  |  |  |  | 99,00 | AGIALNDNFIK | 1188,7 | 1189,7 | 1188,7 | 1189,7 |
|  |  |  |  |  |  |  | 99,00 | AASFNIIPSSTGAAK | 1433,8 | 1434,8 | 1433,8 | 1434,8 |
|  |  |  |  |  |  |  | 96,00 | IKIGINGFGR | 1074,6 | 1075,7 | 1074,6 | 1075,6 |
|  |  |  |  |  |  |  | 76,00 | LVSWYDNEWGYSTR | 1790,8 | 1791,9 | 1790,8 | 1791,8 |
|  | P25861 | G3PC_ANTMA | Glyceraldehyde-3-phosphate dehydrogenase, cytosolic | *Antirrhinum majus* | 2 | 5 | 99,00 | VPTVDVSVVDLTVR | 1497,9 | 1498,9 | 1497,8 | 1498,8 |
|  |  |  |  |  |  |  | 99,00 | AASFNIIPSSTGAAK | 1433,8 | 1434,8 | 1433,8 | 1434,8 |
|  |  |  |  |  |  |  | 99,00 | TLLFGEKPVVVFGR | 1664,0 | 1665,0 | 1663,9 | 1664,9 |
|  |  |  |  |  |  |  | 96,00 | IKIGINGFGR | 1074,6 | 1075,7 | 1074,6 | 1075,6 |
|  |  |  |  |  |  |  | 76,00 | LVSWYDNEWGYSTR | 1790,8 | 1791,9 | 1790,8 | 1791,8 |
|  | P17878 | G3PC_MESCR | Glyceraldehyde-3-phosphate dehydrogenase, cytosolic | *Mesembryanthemum crystallinum* | 2 | 3 | 99,00 | GILGYTEDDLVSTDFIGDNR | 2211,1 | 2212,1 | 2211,0 | 2212,0 |
|  |  |  |  |  |  |  | 99,00 | AASFNIIPSSTGAAK | 1433,8 | 1434,8 | 1433,8 | 1434,8 |
|  |  |  |  |  |  |  | 76,00 | LVSWYDNEWGYSTR | 1790,8 | 1791,9 | 1790,8 | 1791,8 |
| 55 | P21241 | RUBB_BRANA | RuBisCO large subunit-binding protein subunit beta, chloroplastic | *Brassica napus* | 3,57 | 3 | 99,00 | LADLVGVTLGPK | 1181,7 | 1182,7 | 1181,7 | 1182,7 |
|  |  |  |  |  |  |  | 83,00 | IAALKAPGFGER | 1228,7 | 1229,7 | 1228,7 | 1229,7 |
|  |  |  |  |  |  |  | 84,00 | AAVEEGIVVGGGCTLLR | 1699,9 | 1700,9 | 1699,9 | 1700,9 |
| 56 | P08927 | RUBB_PEA | RuBisCO large subunit-binding protein subunit beta, chloroplastic | *Pisum sativum* | 7,29 | 4 | 99,00 | LADLVGVTLGPK | 1181,7 | 1182,7 | 1181,7 | 1182,7 |
|  |  |  |  |  |  |  | 99,00 | LSGGVAVIQVGAQTETELKEK | 2156,1 | 2157,1 | 2156,2 | 2157,2 |
|  |  |  |  |  |  |  | 99,00 | GYISPYFVTDSEK | 1504,7 | 1505,7 | 1504,7 | 1505,7 |
|  |  |  |  |  |  |  | 93,00 | IAALKAPGFGER | 1228,7 | 1229,7 | 1228,7 | 1229,7 |
| 58 | P08927 | RUBB_PEA | RuBisCO large subunit-binding protein subunit beta, chloroplastic | *Pisum sativum* | 11,49 | 6 | 99,00 | LSGGVAVIQVGAQTETELKEK | 2156,1 | 2157,1 | 2156,2 | 2157,2 |
|  |  |  |  |  |  |  | 99,00 | LADLVGVTLGPK | 1181,7 | 1182,7 | 1181,7 | 1182,7 |
|  |  |  |  |  |  |  | 99,00 | TNDLAGDGTTTSVVLAQGLIAEGVK | 2429,2 | 2430,2 | 2429,3 | 2430,3 |
|  |  |  |  |  |  |  | 99,00 | KLRVEDALNATK | 1356,8 | 1357,8 | 1356,8 | 1357,8 |
|  |  |  |  |  |  |  | 99,00 | SAENSLYVVEGMQFDR | 1859,8 | 1860,8 | 1859,8 | 1860,8 |
|  |  |  |  |  |  |  | 94,00 | IAALKAPGFGER | 1228,7 | 1229,7 | 1228,7 | 1229,7 |
| 63 | P12628 | MAOX_PHAVU | NADP-dependent malic enzyme | *Phaseolus vulgaris* | 4,02 | 2 | 99,00 | AIKPTVLIGSSGAGK | 1473,8 | 1474,8 | 1473,8 | 1474,8 |
|  |  |  |  |  |  |  | 99,00 | SIQVIVVTDGER | 1314,7 | 1315,7 | 1314,7 | 1315,7 |
|  | P16243 | MAOC_MAIZE | NADP-dependent malic enzyme, chloroplastic | *Zea mays* | 3,4 | 2 | 99,00 | GLLPPAVLSQELQIK | 1636,9 | 1637,9 | 1636,9 | 1637,9 |
|  |  |  |  |  |  |  | 96,00 | AYELGLATRLPPPSDLVK | 1939,1 | 1940,1 | 1939,1 | 1940,1 |
|  | P22178 | MAOC_FLATR | NADP-dependent malic enzyme, chloroplastic | *Flaveria trinervia* | 1,52 | 2 | 97,00 | QYQVPLQR | 1030,6 | 1031,6 | 1030,6 | 1031,6 |
|  |  |  |  |  |  |  | 94,00 | QYQVPLQR | 1013,5 | 1014,5 | 1013,5 | 1014,5 |
| 68 | P26301 | ENO1_MAIZE | Enolase 1 OS=Zea mays | *Zea mays* | 4,21 | 3 | 99,00 | LTDEIGQKVQIVGDDLLVTNPTR | 2524,3 | 2525,3 | 2524,3 | 2525,3 |
|  |  |  |  |  |  |  | 99,00 | VNQIGSVTESIEAVR | 1600,8 | 1601,8 | 1600,8 | 1601,8 |
|  |  |  |  |  |  |  | 39,00 | FRAPVEPY | 977,5 | 978,5 | 977,5 | 978,5 |
| 73 | P12628 | MAOX_PHAVU | NADP-dependent malic enzyme | *Phaseolus vulgaris* | 4,11 | 3 | 99,00 | SIQVIVVTDGER | 1314,7 | 1315,7 | 1314,7 | 1315,7 |
|  |  |  |  |  |  |  | 99,00 | AIKPTVLIGSSGAGK | 1473,8 | 1474,8 | 1473,8 | 1474,8 |
|  |  |  |  |  |  |  | 23,00 | RPQGLYISLK | 1173,7 | 1174,7 | 1173,7 | 1174,7 |
|  | P22178 | MAOC_FLATR | NADP-dependent malic enzyme, chloroplastic | *Flaveria trinervia* | 2 | 1 | 99,00 | QYQVPLQR | 1030,6 | 1031,6 | 1030,6 | 1031,6 |
| 75 | P32289 | GLNA_VIGAC | Glutamine synthetase nodule isozyme | *Vigna aconitifolia* | 4,83 | 3 | 99,00 | ITEIAGVVLSFDPKPIK | 1826,0 | 1827,0 | 1826,1 | 1827,1 |
|  |  |  |  |  |  |  | 99,00 | HKEHIAAYGEGNER | 1609,8 | 1610,8 | 1609,8 | 1610,8 |
|  |  |  |  |  |  |  | 83,00 | SLLSDLINLNLSDTTEK | 1931,0 | 1932,0 | 1931,0 | 1932,0 |
|  | P04771 | GLNA2_PHAVU | Glutamine synthetase PR-2 | *Phaseolus vulgaris* | 1,77 | 2 | 96,00 | TLPGPVDDPAKLPK | 1462,8 | 1463,8 | 1462,8 | 1463,8 |
|  |  |  |  |  |  |  | 89,00 | FSWGVANR | 935,5 | 936,5 | 935,5 | 936,5 |
| 88 | P34066; O23712 | PSA1A_ARATH; PSA1B_ARATH | Proteasome subunit alpha type-1-A; Proteasome subunit alpha type-1-B | *Arabidopsis thaliana* | 7,22 | 4 | 99,00 | KIFKVDDHIGVAIAGLTADGR | 2195,2 | 2196,2 | 2195,2 | 2196,2 |
|  |  |  |  |  |  |  | 99,00 | IFKVDDHIGVAIAGLTADGR | 2067,1 | 2068,1 | 2067,1 | 2068,1 |
|  |  |  |  |  |  |  | 94,00 | LFQVEYAMEAVK | 1458,7 | 1459,7 | 1458,7 | 1459,7 |
|  |  |  |  |  |  |  | 99,00 | VDDHIGVAIAGLTADGR | 1678,9 | 1679,9 | 1678,9 | 1679,9 |
|  | P52428 | PSA1_ORYSJ | Proteasome subunit alpha type-1 | *Oryza sativa subsp. japonica* | 2 | 3 | 99,00 | NQYDTDVTTWSPAGR | 1709,8 | 1710,8 | 1709,8 | 1710,8 |
|  |  |  |  |  |  |  | 94,00 | LFQVEYAMEAVK | 1458,7 | 1459,7 | 1458,7 | 1459,7 |
|  |  |  |  |  |  |  | 85,00 | NQYDTDVTTWSPAGR | 1741,8 | 1742,8 | 1741,8 | 1742,8 |
| 97 | O23715 | PSA3_ARATH | Proteasome subunit alpha type-3 OS=Arabidopsis thaliana | *Arabidopsis thaliana* | 4,01 | 2 | 99,00 | VFQIEYAAK | 1067,6 | 1068,6 | 1067,6 | 1068,6 |
|  |  |  |  |  |  |  | 99,00 | SSIGTGYDLSVTTFSPDGR | 2000,9 | 2001,9 | 2000,9 | 2001,9 |
| 113 | Q9LSU3 | PSA6_ORYSJ | Proteasome subunit alpha type-6 | *Oryza sativa subsp. japonica* | 7,06 | 5 | 96,00 | AGVTSIGVR | 929,6 | 930,6 | 929,5 | 930,5 |
|  |  |  |  |  |  |  | 82,00 | LYQVEYAFK | 1155,6 | 1156,6 | 1159,6 | 1160,6 |
|  |  |  |  |  |  |  | 99,00 | ATEIEVGVVR | 1071,6 | 1072,6 | 1071,6 | 1072,6 |
|  |  |  |  |  |  |  | 99,00 | LYQVEYAFK | 1159,6 | 1160,6 | 1159,6 | 1160,6 |
|  |  |  |  |  |  |  | 97,00 | ALTTEEIDQHLTAISERD | 2056,0 | 2057,0 | 2056,0 | 2057,0 |
|  | O48551 | PSA6_SOYBN | Proteasome subunit alpha type-6 | *Glycine max* | 1,91 | 4 | 99,00 | ATSAGLKDQEAINFLEKK | 1976,1 | 1977,1 | 1976,0 | 1977,0 |
|  |  |  |  |  |  |  | 99,00 | ATEIEVGVVR | 1071,6 | 1072,6 | 1071,6 | 1072,6 |
|  |  |  |  |  |  |  | 72,00 | LFQVEYAFK | 1159,6 | 1160,6 | 1159,6 | 1160,6 |
|  |  |  |  |  |  |  | 72,00 | YLGLLATGMTADAR | 1467,7 | 1468,7 | 1467,7 | 1468,7 |
