# Supplementary figures and images for: Shoot differentiation from protocorm callus cultures of Vanilla planifolia (Orchidaceae): proteomic and metabolic responses at early stage
Source: BMC Plant Biol. 2010 May 5;10:82. doi: 10.1186/1471-2229-10-82 (PMC3095354; doi:10.1186/1471-2229-10-82)

C A4 d15

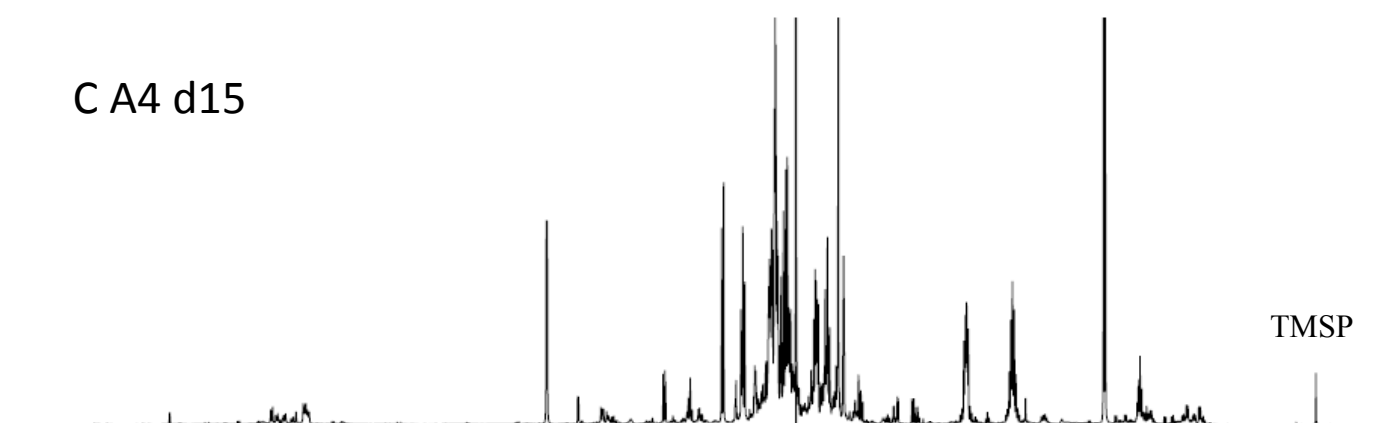

C A10 d15

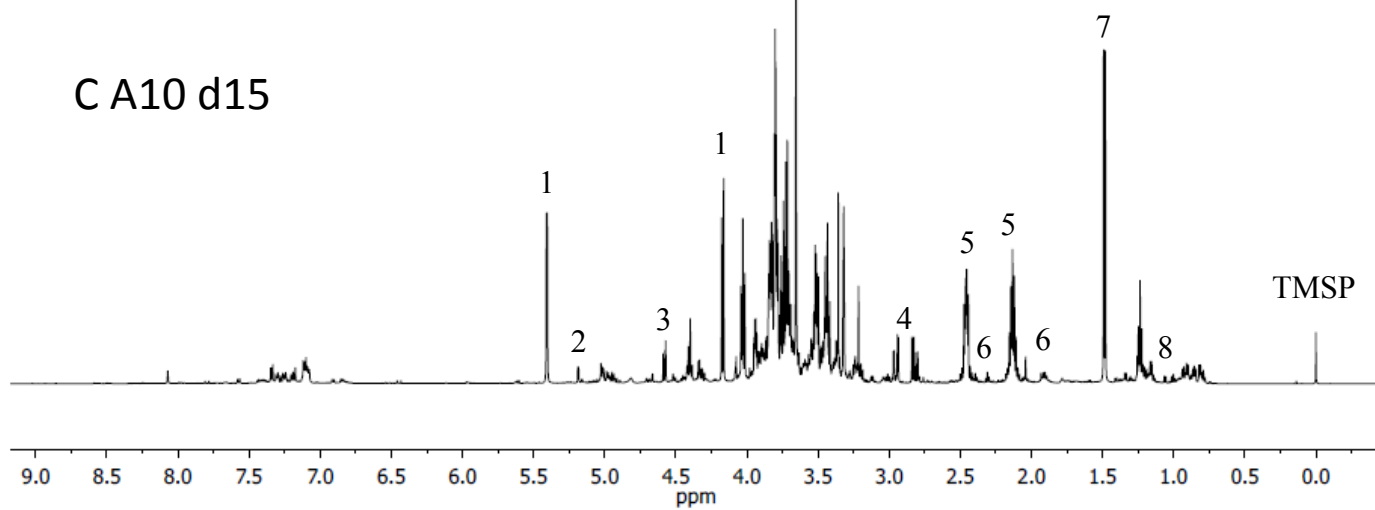

Supplement: Additional file 3 — 1H NMR spectra (methanol-d4-KH2 PO4 in D2 O, pH 6.0 extract) of CA4 d15 and CA10 d15 calli in the range of δ -0.5 - 9.0. Visual and comparative inspection of CA4 d15 and CA10 d15 calli spectra. Assignments: 1, sucrose; 2, α-glucose; 3, β-glucose; 4, asparagine; 5, glutamine; 6, δ-aminobutyric acid; 7, alanine; 8, valine. [file 1471-2229-10-82-S3.PDF]

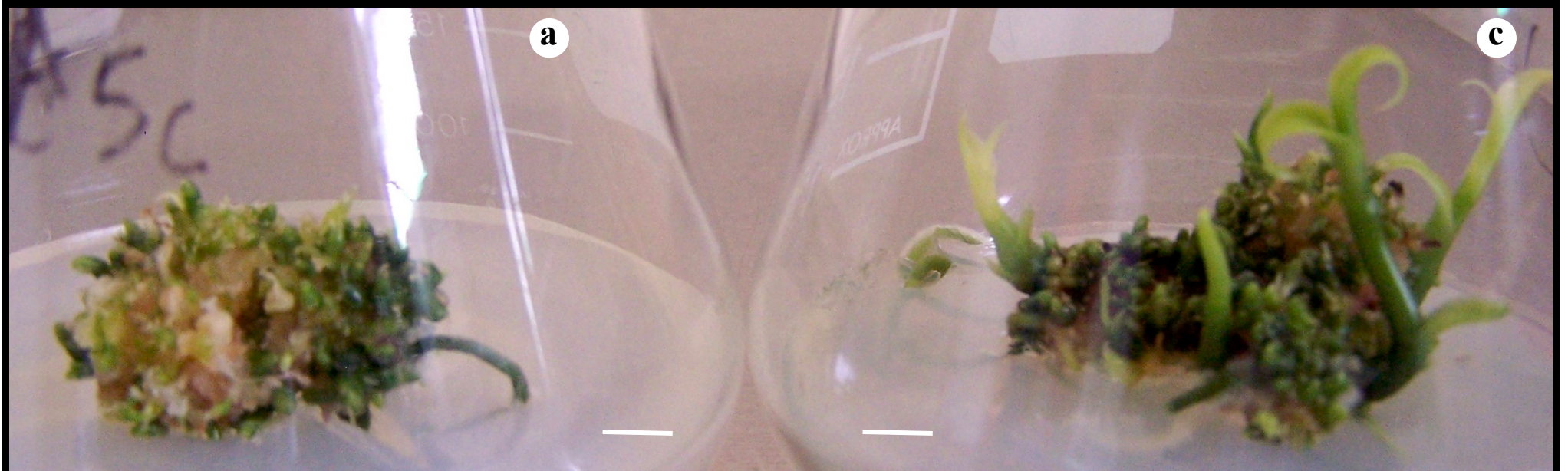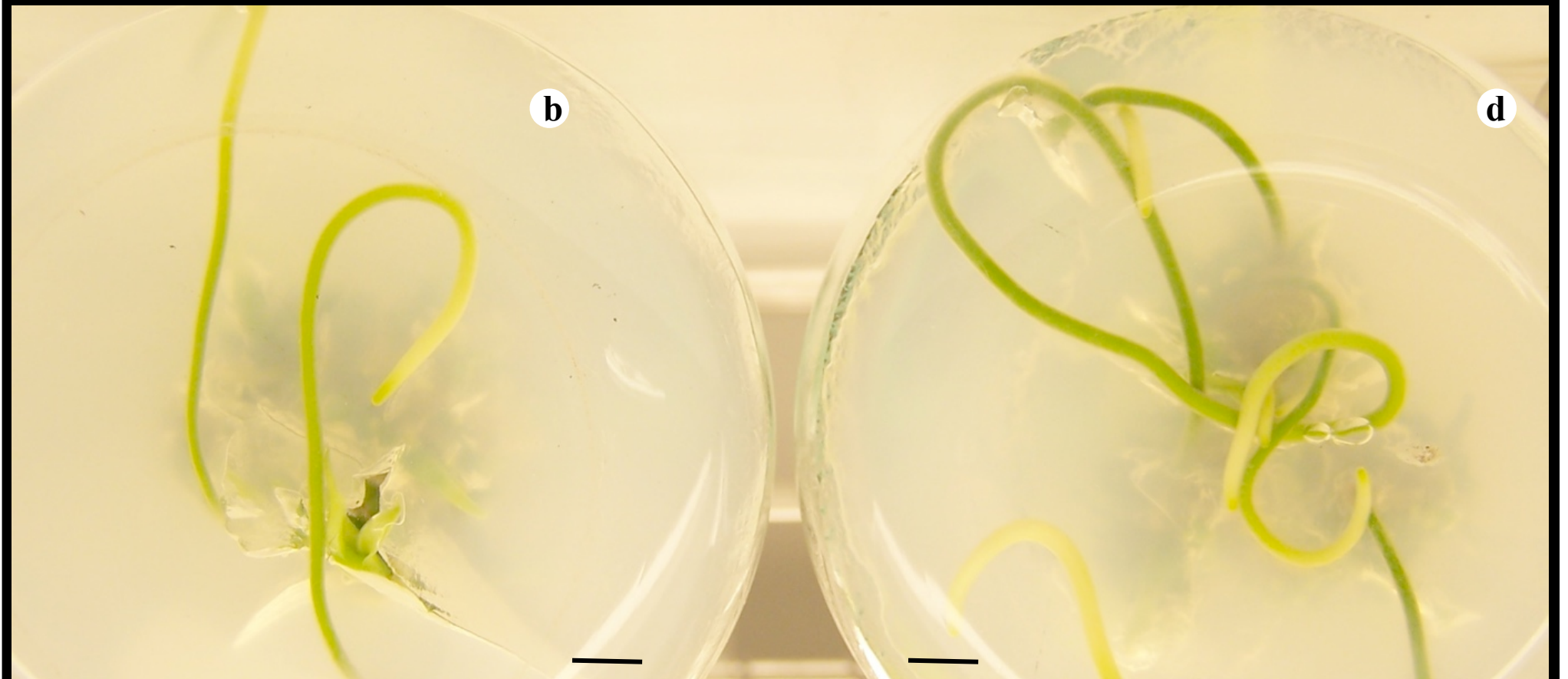

Supplement: Additional file 4 — PLBs, and roots formation from embryogenic/organogenic callus after 120 days culture in A5 medium and A10 medium. Proliferating PLBs with well-developed roots on embryogenic/organogenic callus cultured on two types of shoot regeneration medium. a and b Embryogenic/organogenic callus in A5 medium: BM. c and d Embryogenic/organogenic callus in A10 medium: BM + NAA 0.5 mg l-1 (CA10 d120 callus). Bars in a and c = 7.75 mm; bars in b and d = 5.6 mm. [file 1471-2229-10-82-S4.PDF]
