# Supplementary material for: Shoot differentiation from protocorm callus cultures of Vanilla planifolia (Orchidaceae): proteomic and metabolic responses at early stage
Source: BMC Plant Biol. 2010 May 5;10:82. doi: 10.1186/1471-2229-10-82 (PMC3095354; doi:10.1186/1471-2229-10-82)
Supplement: Additional file 5 — Morphogenic response of embryogenic callus cultures or Protocorm-like body (PLB) - derived plantlets of V. planifolia after transferring on different media. Preliminary experiments to choose the best medium at each step of the procedure of plantlet regeneration from callus derived protocorm of V. planifolia. [file 1471-2229-10-82-S5.DOC]

| Number of medium (name of medium) | Plant growth regulators in basal medium (BM) | Callus maintenance and proliferation | Shoot differentiation and PLB formation | PLB elongation | PLB -derived Plantlet rooting |
| --- | --- | --- | --- | --- | --- |
| 1 (A1) | 0.5mg l^-1^ NAA + 0.5 mg l^-1^ GA | + | + | + | NT |
| 2 (A2) | 0.5 mg l^-1^ IAA + 0.5 mg l^-1^ GA | + | + | + | NT |
| 3 (A3) | 0.5 mg l^-1^NAA + 0.3 mg l^-1^ TDZ | ++++ | 0 | NT | NT |
| 4 (A4) | 0.5 mg l^-1^IAA + 0.3 mg l^-1^ TDZ | +++++ | 0 | NT | NT |
| 5 (A5) | None | ++ | +++++ | ++++ | ++++ |
| 6 (A6) | 0.5 mg l^-1^IAA + 0.3 mg l^-1^ TDZ + 0.5 mg l^-1^ GA | ++ | + | + | NT |
| 7 (A7) | 1 mg l^-1^NAA | ++ | ++ | 0 | ++ |
| 8 (A8) | 1 mg l^-1^ IAA | ++ | ++ | + | +++ |
| 9 (A9) | 0.5 mg l^-1^IAA + 0.5 mg l^-1^ NAA | ++ | + | + | +++ |
| 10 (A10) | 0.5 mg l^-1^ NAA | +++ | +++++ | +++++ | ++++ |

**Additional file 5. Morphogenic response of embryogenic callus cultures or Protocorm - like body (PLB) – derived plantlets of *V. planifolia* after transferring on different media.** These media contained basal medium (BM) supplemented with or without plant regulators. Maximum response is indicated by +++++ and minimum by +; 0 represents no response; NT means not tested.
