# Supplementary material for: Shoot differentiation from protocorm callus cultures of Vanilla planifolia (Orchidaceae): proteomic and metabolic responses at early stage
Source: BMC Plant Biol. 2010 May 5;10:82. doi: 10.1186/1471-2229-10-82 (PMC3095354; doi:10.1186/1471-2229-10-82)
Supplement: Additional file 6 — Appearance of embryogenic/organogenic calli cultivated onto A4 medium and A10 medium. Comparison of morphologic evolution of organogenic callus on A4 medium and A10 medium during 120 days without subculture. [file 1471-2229-10-82-S6.DOC]

**Additional file 6. Appearance of embryogenic/organogenic calli cultivated onto A4 medium and A10 medium.** After 15 days, there were no visible differences between the CA4 and CA10 calli.

| Observation times | Embryogenic/organogenic calli on A4 medium (CA4) | Embryogenic/organogenic calli on A10 medium (CA10) |
| --- | --- | --- |
| 15 days | Calli | Calli |
| 20 days | Calli* | Calli with yellow regions |
| 30 days | Calli* with yellow and pale green regions | Calli with pale green or green shoot primordia : early stage of protocorm-like body (PLB) development |
| 60 days | Calli with yellow-green regions | Calli with yellow-green PLBs and green-differentiated PLBs wearing leaf sheath |
| 75 days | Calli with yellow-green regions | Calli with yellow-green PLBs and green-differentiated PLBs; root primodia emerging |
| 90 days | Calli with pale green and poorly-differentiated structures; some regions started to turn brown | Callus with green and deep green PLBs; regeneration of one or two roots |
| 120 days | Calli with pale green and poorly-differentiated structures; necrosis was observed | Calli with very differentiated PLBs, PLB-derived plantlets and well-developed roots |

* After 20 days, 20 - 30% of organogenic calli exhibited white nodular compact structures.
